# Highly-Oriented Polylactic Acid Fiber Reinforced Polycaprolactone Composite Produced by Infused Fiber Mat Process for 3D Printed Tissue Engineering Technology

**DOI:** 10.3390/polym17152138

**Published:** 2025-08-05

**Authors:** Zhipeng Deng, Chen Rao, Simin Han, Qungui Wei, Yichen Liang, Jialong Liu, Dazhi Jiang

**Affiliations:** School of Materials, Sun Yat-sen University, Shenzhen Campus, Shenzhen 518107, China

**Keywords:** PLA fiber mat, PCL composite, 3D print process, orientation angle, mechanical properties, vacuum assisted resin infusion

## Abstract

Three-dimensional printed polycaprolactone (PCL) tissue engineering scaffolds have drawn increasing interest from the medical industry due to their excellent biocompatibility and biodegradability, yet PCL’s poor mechanical performance has limited their applications. This study introduces a biocompatible and biodegradable polylactic acid (PLA) fiber reinforced PCL (PLA/PCL) composite as the filament for 3D printed scaffolds to significantly enhance their mechanical performance: Special-made PLA short fiber mat was infused with PCL matrix and rolled into PLA/PCL filaments through a “Vacuum Assisted Resin Infusion” (VARI) process. The investigation revealed that the PLA fibers are highly oriented along the printing direction when using this filament for 3D printing due to the unique microstructure formed during the VARI process. At the same PLA fiber content, the percentage increase in Young’s modulus of the 3D printed strands using the filaments produced by the VARI process is 127.6% higher than the 3D printed strands using the filaments produced by the conventional melt blending process. The 3D printed tissue engineering scaffolds using the PLA/PCL composite filament with 11 wt% PLA fiber content also achieved an exceptional 84.2% and 143.3% increase in peak load and stiffness compared to the neat PCL counterpart.

## 1. Introduction

Every year, millions of patients undergo varying degrees of tissue damage and organ failure as a result of diseases, accidental injuries, and declines in physical function. Although clinical practices have developed autologous and allogeneic transplantation procedures for patient treatment, they frequently encounter a range of issues, including immune rejection and potential risks of complications. With the progress of biomedical and engineering technologies, a series of artificial tissues and organs designed and developed based on tissue engineering strategies have been applied in clinical treatments, such as bone, cartilage, tendons and ligaments, cardiovascular systems, blood vessels, and skin [1,2]. Polycaprolactone (PCL) has been widely adopted in tissue engineering due to its excellent biocompatibility, biodegradability, non-toxicity, thermal stability, and ease of processing [3]. PCL can be used to fabricate tissue implants with interconnected porous structures through FDM 3D printing [4,5,6,7,8]. These PCL implants were printed with high porosity to facilitate the transportation of oxygen and nutrients for accelerating tissue repair and regeneration [9]. However, the 3D printed porous structure reduces the strength and stiffness of the implants, and since PCL’s strength and modulus are also low, this has limited 3D printed PCL application in tissue engineering [10].

To enhance the mechanical performance of PCL, researchers devoted considerable efforts to develop PCL-based composites through nanofiller incorporation [11,12,13,14], polymer blending [15,16,17,18,19,20,21], and fiber reinforcement [22,23,24,25,26,27,28,29,30,31]. Among these methods, preparing PCL composites via pure melt blending is a low-cost and efficient approach. For example, Jon et al. [32] prepared PLA/PCL blends with different proportions by mixing PLA and PCL using a twin-screw extruder. The results showed that all blends are immiscible; therefore, compatibilizers often need to be added. However, this may affect biocompatibility. In contrast, reinforcing PCL with polylactic acid (PLA) fiber stands out as an effective solution because PLA fiber is stronger and stiffer than PCL, whilst it is also biodegradable and biocompatible [25,33,34]. In addition, studies have shown that PLA fibers can regulate the degradation rate of PCL composites. Ju et al. [22] prepared PLA fiber/PCL composite films through melt blending and hot pressing. The enzymatic degradation assays revealed that PLA fibers partially impeded PCL degradation. Scaffaro et al. [35] prepared PLA/PCL co-mingled nanofibrous mats via electrospinning and investigated their degradation behavior in buffer solutions at varying pH. The pH significantly influenced degradation kinetics: elevated PLA content decelerated degradation at pH 4 or 7, whereas at pH 10, higher PLA content accelerated degradation. Consequently, it is anticipated that PLA fiber-reinforced PCL (PLA/PCL) composites will possess superior mechanical performance for tissue engineering applications.

The main fabrication methods for PLA/PCL composites include melt blending [22], melt blending with in situ nanofibrillation [10,23,24], fiber winding [25], electrospinning with hot-pressing [26,27], yarn weaving with hot-pressing [36], and electrospinning [29,37,38,39,40]. Ju et al. fabricated PLA/PCL composite sheets through the melt blending process. PCL and 10 wt% PLA fibers were heated up and blended by a pair of mechanical screws, followed by hot-pressing. The tensile strength and Young’s modulus of the PLA/PCL composite were 7% and 42% higher than the neat PCL [22]. Kelnar et al. produced a PLA/PCL composite with 20 wt% PLA fiber content through the melt-drawn process and reported a 25% increase in tensile strength compared to the neat PCL [10]. Kakroodi et al. fabricated PLA/PCL composite films through a hot pressing process. Although the mechanical performance of the film was improved significantly, the bulk composite was not produced [23]. Guarino et al. immersed PLA fibers in PCL solution with toxic solvents, followed by fiber winding, impregnation, and a drying process to fabricate the PLA/PCL composite [25]. Chen et al. prepared PLA and PCL co-spun fiber mats via electrospinning, followed by hot pressing with additional PCL sheets, and reported that the tensile strength of the composite was increased by 27% with 15 wt% fiber content; however, the process also adopted toxic solvents (chloroform and N, N-dimethylformamide) [26].

To the authors’ knowledge, there have been few reports on fabricating PLA fiber reinforced PCL composites without using toxic solvents, and the improvements in the mechanical performance of PLA/PCL were rather low [10,22,25,26]. Various solvent systems employed in the preparation of PLA/PCL composites are summarized in Table 1. There have been no reports investigating the PLA/PCL composite filament for 3D printed tissue engineering scaffolds either. Therefore, it is necessary to develop a non-toxic process to produce PLA/PCL composite filament with higher mechanical performance and investigate their mechanical performance when they are adopted as the filament for FDM 3D printing.

This study introduces a new fabrication approach that produces PLA/PCL composites as the filament material for 3D printed parts with significantly enhanced mechanical performance. PLA/PCL filaments produced by the conventional melt blending processes were also adopted as references. The fiber orientation and damage mechanism of the PLA/PCL composites were also investigated with the aid of a scanning electron microscope.

## 2. Materials and Methods

### 2.1. Material Preparation

PCL pellets with a density of 1.02 g/cm^3^ supplied by Perstorp, Malmö, Sweden, were used as the matrix material. Short PLA fibers, made from L-polylactic acid with a linear density of 1.7 dtex, supplied by ADVANSA GmbH, Hamm, Germany, were used as the fiber material. The PLA fibers have an average length of 5.06 ± 0.03 mm and a diameter of 13.05 ± 0.78 µm. The PLA fibers exhibited a tensile strength of 412.5 MPa, a Young’s modulus of 5120 MPa, and a fracture strain of 42%.

**Figure 1:**
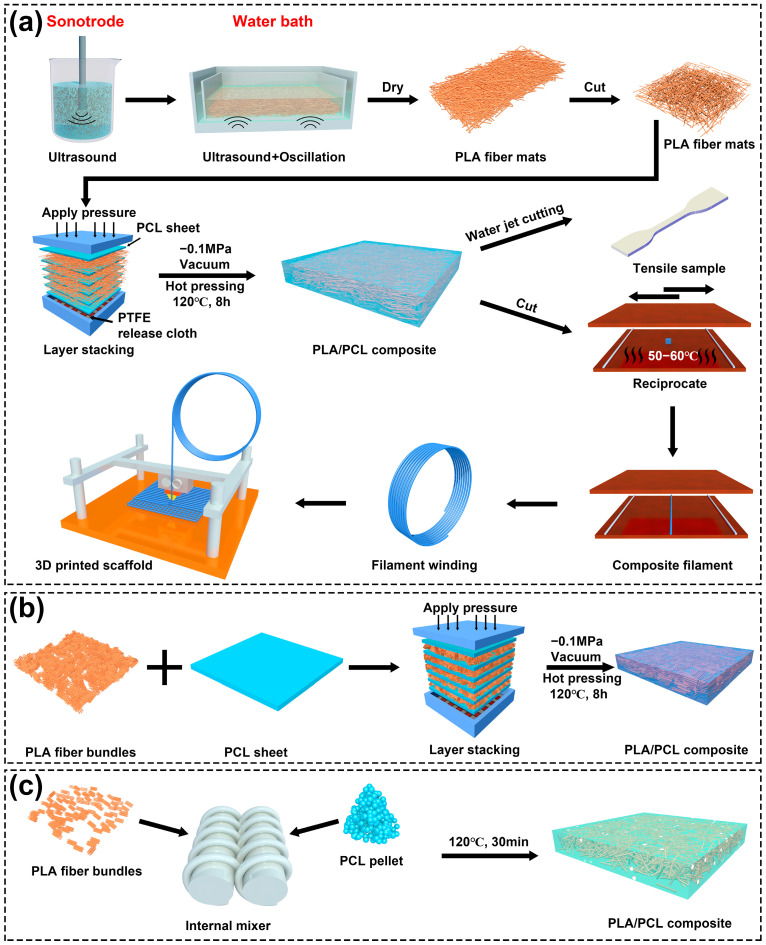
Schematic illustration of (**a**) VARI process and process to adopt the PLA/PCL composite for FDM printing, (**b**) conventional hot-pressing process, and (**c**) melt blending process.

As shown in Figure 1, this study introduces a “vacuum assisted resin infusion” (VARI) process to produce the filament for 3D printing: the PLA/PCL composite was fabricated by embedding the PCL in a pre-made PLA fiber mat assisted by vacuum, heat, and pressure, followed by rolling the composite into filaments. PLA fiber bundles were dispersed in deionized water for 6 min using a sonotrode-type ultrasonicator (20 kHz, 600 W), with the mass ratio of fibers to water being 1:250. The mixture was then laid flat in a container in a bath-type ultrasonicator (40 kHz, 200 W) for 6 min with simultaneous shaking to produce a mat with uniformly dispersed PLA fibers. The deionized water in the mat was drained through filtration, and the mat was rinsed with ethanol and drained again. Subsequently, the PLA fiber mat was dried in a vacuum oven overnight at 45 °C. PCL pellets were pressed into sheets with a thickness of 0.5 mm at 90 °C and 10 MPa using a hot-press machine (TY-305F-200KN, Ningbo Tianyu Machinery Equipment Co., Ltd., Ningbo, China). The PCL sheets and PLA fiber mats were then laid alternately and placed in a stainless-steel mold with a depth of 3 mm. The mold was then placed into a vacuum oven and heated to 120 °C over 8 h while 30 kPa of pressure was applied. The composite plate was then cooled for over 2 h and taken out of the mold. The processing temperature (120 °C) was selected based on differential scanning calorimeter (DSC) characterization of raw materials, as shown in Figure S1: it exceeds the melting onset of PCL (49.43 °C) to ensure matrix flow; however, it remains below the melting peak of PLA fibers (169.63 °C) to preserve fiber integrity. PLA/PCL composites with different fiber contents were produced as shown in Table 2. PLA/PCL composites were then placed on a heated metal plate at 60 °C and rolled into filament with a diameter of 1.75 ± 0.10 mm for the FDM 3D printer. In addition, PLA/PCL composites with 11 wt% PLA fiber content were produced by the conventional hot-pressing and melt blending process, as shown in Figure 1b,c as a reference. With the aid of optical microscopes, it was observed that the fiber length was not reduced after the melt blending process (Figure S2).

Three-layered 3D constructs adopted from Huang et al. were printed using the filaments produced in this study [41]. The thickness of each printed layer was 0.6 mm, and the distance of the adjacent path was 1.2 mm with 0°/90° lay-down patterns. The nozzle diameter and printing velocity were 0.8 mm and 4 mm/s, respectively. The nozzle and platform temperatures were 155 °C and 35 °C, respectively.

### 2.2. Composites and 3D Printed Component Characterization

The morphology of composites fabricated through different preparation processes was observed using a polarized light microscope (PLM, MSD460, Murzider Technology Co., Ltd., Dongguang, China) and a scanning electron microscope (SEM, SU5000, Hitachi, Tokyo, Japan) to detect defects such as voids. For the composites, SEM observation was conducted on both the tensile fracture surfaces and brittle fracture surfaces obtained via liquid nitrogen freezing. For filaments and 3D printed strands, the specimens were placed side by side and cut at an angle of 5° with respect to the axial direction of the specimens to obtain oblique cross-sections. The fiber orientation was qualitatively analyzed through SEM observation. Additionally, the specimens were cut at a 90° angle to their axial direction to obtain cross-sections. The lengths of the major and minor axes of the fiber profiles in the cross-sections were measured using SEM observation combined with Nano Measurer software (Version 1.2.0). Through statistics and calculations (detailed calculation seen in Figure S4), the distribution of fiber orientation angles was quantitatively analyzed. For each sample, 280 to 300 fibers were statistically analyzed. The tensile properties of the composites and the 3D printed strands/parts were also tested.

**Figure 2:**
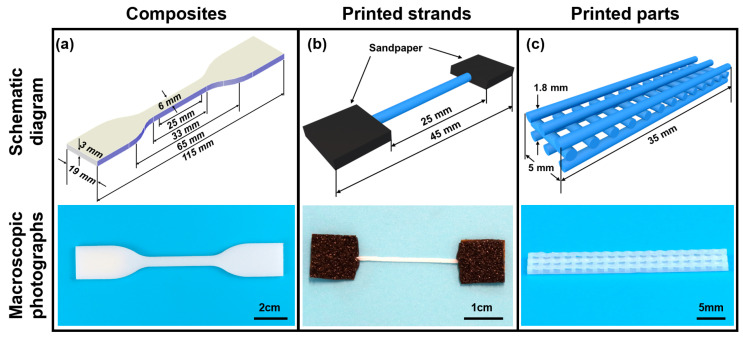
Specimens for tensile test, including (**a**) the composites, (**b**) 3D printed strands, and (**c**) 3D printed tissue engineering scaffolds.

The tensile properties of the composites were tested based on ASTM D638-22 [42]. Composite plates produced in Section 2.1 were cut into specimens (Figure 2a) through a water jet. The stress–strain curves of the specimens were obtained using a universal mechanical testing machine (LE3104, Lishi Instruments Co., Ltd., Shanghai, China) with a loading rate of 50 mm/min. An extensometer was utilized to measure the elastic modulus (Young’s modulus) of the specimens.

As shown in Figure 2b, 3D printed strands were tested based on the method reported by Haque et al. [43]. Individual strands with 45 × 0.8 mm in length and width were printed. Tensile tests were conducted using the same mechanical testing machine and loading rate, and the stress–strain curves of the specimens were obtained. As shown in Figure 2c, 3D printed tissue engineering scaffolds were tested based on the method reported by Huang et al. [41], and parts with 35 × 5 × 1.8 mm in length, width, and height were printed. Tensile tests were conducted using the same mechanical testing machine and loading rate, and the load–displacement curves of the specimens were obtained. At least five specimens for each type were tested for consistency.

All values in this study were reported as mean ± standard deviation (SD). The difference among groups was determined by one-way analysis of variance (ANOVA) and Tukey’s test, when *p* < 0.05 (* means *p* < 0.05, ** means *p* < 0.01, and *** means *p* < 0.001) was considered to be statistically significant. NS indicates not significant.

## 3. Results and Discussion

### 3.1. Defect Inspection

**Figure 3:**
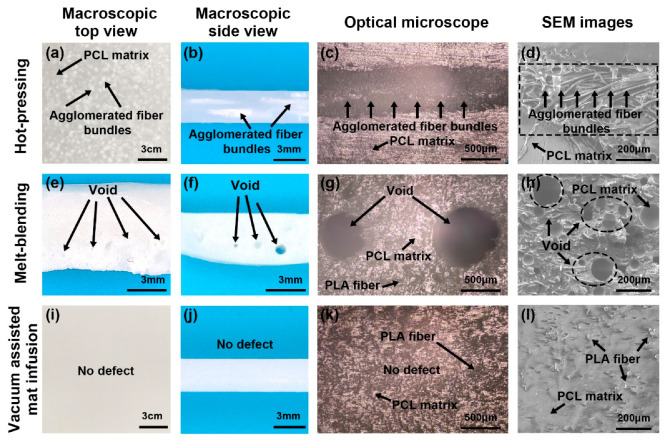
Images of composites fabricated using different methods at different scales. Macroscopic top view (**a**), macroscopic side view (**b**), optical microscope image (**c**) and SEM image (**d**) of PLA/CPL composites prepared through the hot pressing; Macroscopic top view (**e**), macroscopic side view (**f**), optical microscope image (**g**) and SEM image (**h**) of PLA/CPL composites prepared through the melt-blending; Macroscopic top view (**i**), macroscopic side view (**j**), optical microscope image (**k**) and SEM image (**l**) of PLA/CPL composites prepared through the VARI process.

Figure 3 shows the images of the PLA/PCL produced by the hot-press, melt blending, and VARI process. For the conventional hot-press process, agglomerated PLA fiber bundles were observed in the composite because of the poor dispersion of fibers, as shown in Figure 3a–c [44]. Most portions of the PCL lacked fiber reinforcement because most PLA fibers were bundled together. PCL also cannot infiltrate into the tightly packed fiber bundles, as shown in Figure 3d. Since the agglomerated PLA fiber bundles may also block the printing nozzle, the hot-pressed composite filament is not suitable for 3D printing.

Agglomerated fiber bundles were not observed in the PLA/PCL composite produced through melt blending. However, voids of varying sizes resembling trapped air were observed as shown in Figure 3e–h. This is probably because the trapped air within the fiber bundle was unable to escape from the composite during the blending process. The viscosity of the mixture was also significantly increased due to the addition of fibers, which further prevented the air from escaping.

**Figure 4:**
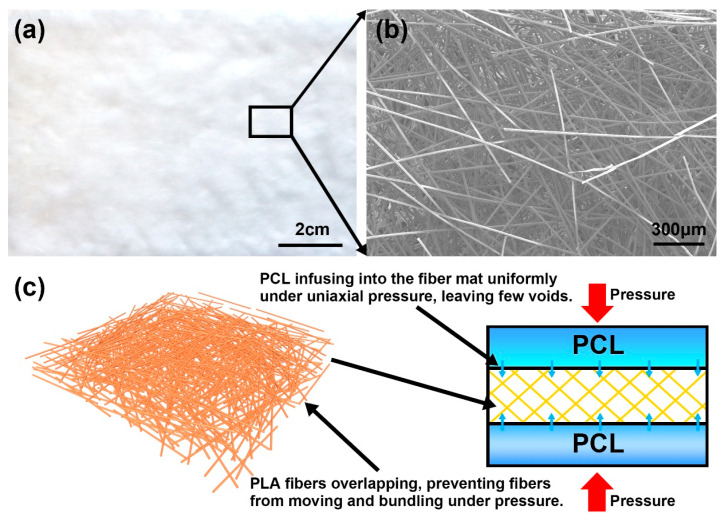
(**a**) Macroscopic photographs of PLA fiber mats. (**b**) SEM images of PLA fiber mats. (**c**) Schematic diagram of PCL infusion into PLA fiber mats.

In contrast, no agglomerated fiber bundles nor voids were observed in the PLA/PCL composite prepared through the VARI process, as shown in Figure 3i–l. As shown in Figure 4a,b, the fibers were well distributed and overlapped with one another in the PLA fiber mat. During the vacuum-assisted heating and pressing process, the overlap creates enough interaction between the fibers, thus preventing them from moving and bundling together. As shown in Figure 4c, the mat was subjected to uniform pressure from the top and bottom, whilst the gap between the fibers left wide passes for the molten PCL. This allows the molten PCL to infiltrate into the PLA fiber mat uniformly and smoothly; therefore, few voids were formed in the composite. As a result, the PLA/PCL composite prepared using the VARI process exhibits good fiber distribution and few defects.

### 3.2. Mechanical Performance of PLA/PCL Composites

**Figure 5:**
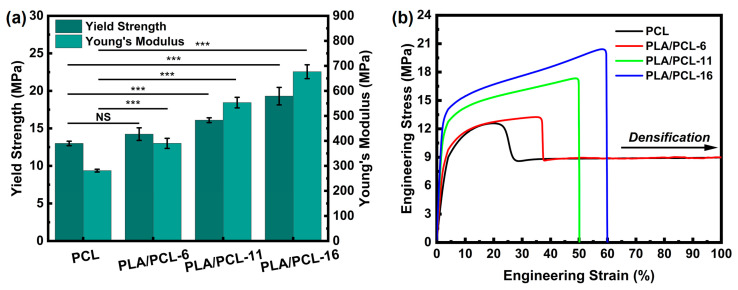
(**a**) Yield strength and Young′s modulus and (**b**) tensile stress−strain curves of PCL and PLA/PCL composites through the VARI process. NS indicates not significant, *** *p* < 0.001.

**Figure 6:**
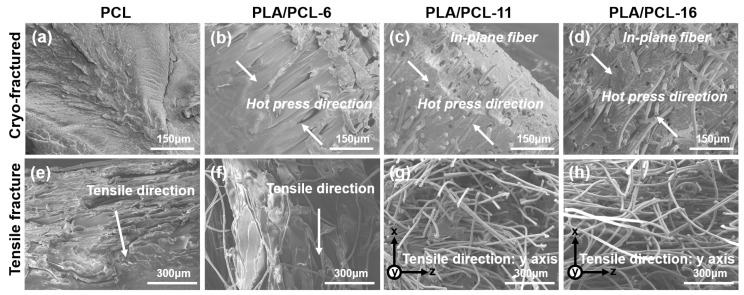
(**a**–**d**) SEM images of the cryo-fractured surface of composites. (**e**–**h**) SEM images of the tensile fractured surface of composites.

The mechanical performance of PLA/PCL composites prepared by the VARI process was tested. As shown in Table 3 and Figure 5, the yield strength and Young’s modulus of the composite gradually increase as the fiber content increases. The error bars in Figure 5a indicate that the composites prepared using the VARI process exhibited good consistency in mechanical performance, which was attributed to the uniformly distributed fiber and fewer defects within the composite. Compared with pure PCL, there was no significant difference in the yield strength of the composite material with a fiber content of 6 wt%, while all the others showed significant differences (*p* < 0.001). Figure 6 presents the SEM images of the cryo-fracture surfaces of PCL and PLA/PCL composite. The fibers were not only uniformly distributed within the PCL matrix; however, they were also predominantly oriented within the same plane, resulting in higher in-plane performances such as tensile strength and modulus. In addition, the overlapping fiber increased the friction between the polymer chains during tension, thus further increasing the composites’ tensile performance.

As shown in Figure 5b, the tensile stress–strain curves of the neat PCL and PLA/PCL-6 are similar, whereas the PCL/PCL-11 and PLA/PCL-16 are less ductile. When the fiber content is low, the composite undergoes a significant plastic deformation, the matrix undergoes plastic flow, whilst the fibers maintain their rigidity. The plastic flow of the matrix will drive the fibers to align along the tensile direction. As a result, the PLA fibers in PLA/PCL-6 were stretched along with the deforming PCL matrix, and the composite behaves more like the neat PCL (Figure 6e,f).

As the fiber content increases to 11 wt% and above, the tensile fracture surfaces of PLA/PCL-11 and PLA/PCL-16 are relatively smooth and exhibit lesser ductile fracture (Figure 6g,h). This is because the increase in the PLA fiber content increases the stiffness of the composite, which leads to the formation of initial cracks within the matrix. The sustained increase in load leads to the propagation of cracks along the fiber/matrix interface, which, in turn, induces fiber pull-out. This ultimately results in material fracture, imparting a brittle fracture-like behavior to the fracture surface of the composite. As shown in Figure 6g,h, when a fracture occurs, the fibers are pulled out from the matrix rather than deforming with the matrix, as in Figure 6f. Because of the different damage patterns, PLA/PCL-11 and PLA/PCL-16’s stress–strain curves are significantly different from the neat PCL and PCL with 6 wt% PLA fiber content.

It should be noted that although the fracture energy at break (area under the curve) of PCL is higher than PLA/PCL-11 and PLA/PCL-16 (Figure 5b), the fracture energy of these composites is less important than their yield strengths and modulus because the tissue scaffold is considered damaged when permanent deformation occurs.

### 3.3. Morphology and Mechanical Performance of the Printed Strands

PLA/PCL composite with 11 wt% PLA fiber content was selected as the filament for FDM 3D printing due to its high yield strength and Young’s modulus, whilst its fiber content is not too high to potentially block the printing nozzle of the printer used in this study. Neat PCL and PLA/PCL composites produced using the VARI and melt blending processes were adopted as the filament for 3D printing. The filaments produced using the melt blending process were inspected for voids before the 3D printing process, and the sections with voids were removed to eliminate defects.

**Figure 7:**
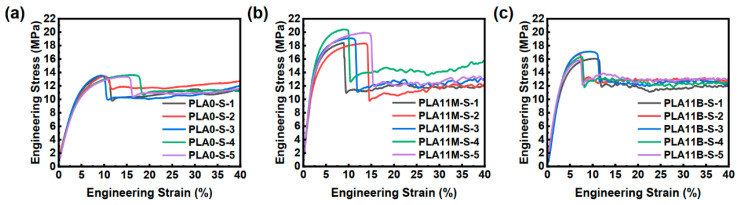
Typical stress–strain curves of 3D printed strands: (**a**) PLA0-S, (**b**) PLA11M-S, and (**c**) PLA11B-S.

The stress–strain curves of the 3D printed strands are shown in Figure 7. The yield strength and Young’s modulus of the strands are listed in Table 4. As shown in Table 4, the yield strength and Young’s modulus of 3D printed PLA/PCL produced by the melt blending process (PLA11B-S) were increased by 21.66% and 65.21%, respectively, compared to the neat PCL (PLA0-S). Interestingly, the yield strength and Young’s modulus of 3D printed PLA/PCL produced by the VARI process (PLA11M-S) were increased by 42.73% and 148.45%. That is to say, despite PLA11M-S and PLA11B-S having the same fiber content, the percentage increase in the strength and modulus of the strands produced by the VARI process is 97.3% and 127.6% higher than their melt blending counterpart. As shown in Figure S3, compared with pure PCL (PLA0-S), both PLA11M-S and PLA11B-S exhibit significant differences in mechanical properties (*p* < 0.001), and there is also a significant difference between PLA11M-S and PLA11B-S (*p* < 0.001).

**Figure 8:**
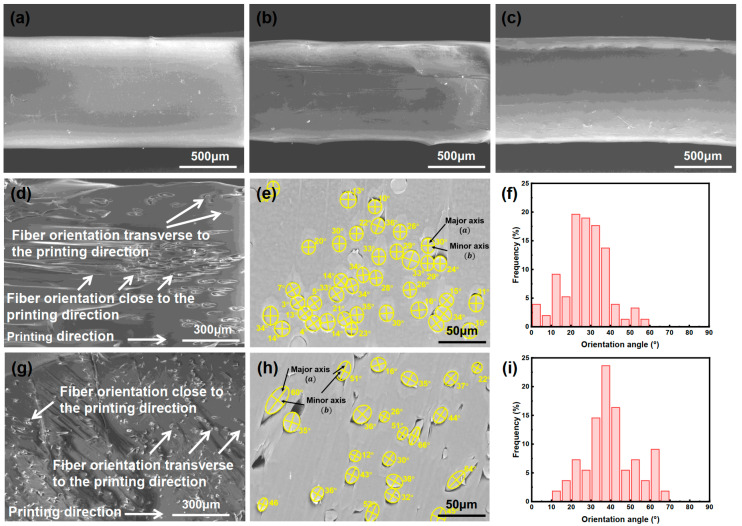
Surface morphology of (**a**) PLA0-S strand, (**b**) PLA11M-S strand, and (**c**) PLA11B-S strand. (**d**) Oblique cross-sectional morphology of PLA11M-S. (**e**) Cross-sectional morphology of PLA11M-S for calculating its fiber orientation distribution; a detailed calculation is shown in Figure S4. (**f**) Orientation angles distribution of PLA11M-S. (**g**) Oblique cross-sectional morphology of PLA11B-S. (**h**) Cross-sectional morphology of PLA11B-S for calculating its fiber orientation distribution, detailed calculation is shown in Figure S4. (**i**) Orientation angles distribution of PLA11B-S.

As shown in Figure 8, there are significantly more fibers aligned closer to the printing direction in the strands produced by the VARI process (PLA11M-S) compared to the strands produced by the melt blending process (PLA11B-S). As shown in Figure 8f,i, there were 59% fibers oriented between 0° and 30° in PLA11M-S, whereas only 18% fibers were oriented between 0° and 30° in PLA11B-S. This explains why Young’s modulus of PLA11M-S is significantly higher than PLA11B-S, despite sharing the same fiber content. This also indicates that the VARI process creates a unique feature that promotes more fibers to align closer to the printing direction.

**Figure 9:**
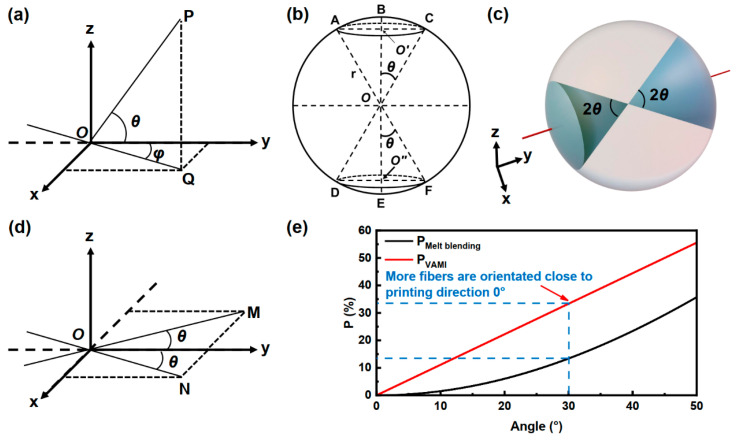
Schematic illustration of the different fiber orientation types’ models. (**a**–**c**) Random orientation for the melt blending process, and (**d**) in-plane orientation for the VARI process. (**e**) The probability of fiber occurrence P as a function of the angle parameter. θ is the angle between the fiber orientation (OP, CD, AF, OM, ON) and the printing direction (y-axis, BE); *φ* is the angle between the projection of the fiber orientation on the xy-plane and the printing direction; O is the center of the sphere, O’ and O’’ are the centers of the bases of cones AOC and DOF respectively; r is the radius of the sphere; ABC and DEF are the spherical region where fibers within θ are located.

By considering the printing direction to be 0°, fibers with their orientation closer to 0° indicate that they are more aligned with the printing direction. When the composite is produced through the melt blending process, the fibers are distributed in a spatially random orientation. Consequently, it can be assumed that fibers exist at various orientations (angles) throughout a 3D space, and a spherical model can be utilized to represent this arbitrary fiber orientation. As illustrated in Figure 9a, consider a fiber with an orientation of OP forming an angle θ with the y-axis (printing direction); its projection on the xy-plane is denoted as OQ, with an angle *φ* relative to the y-axis. The range of θ is from 0° to 90°. For any given angle θ, the surface area projected onto the sphere was $S_{Melt\;blending}$, which comprises the spherical cap surface area (ABC and DEF in Figure 9b). Based on the preceding discussion, as illustrated in Figure 9b,c, assuming the radius of the sphere is r and the surface area of the sphere is $S_{Sphere}=4\pi r^{2}$. When fibers are randomly oriented in space, the probability of fibers appearing at any angle θ, $P_{Melt\;blending}$, can be expressed as Equations (1) and (2):

(1) $$S_{Meltblending}=2\cdot 2\pi \cdot r\cdot \left(r-r\cdot \cos\theta \right)=4\pi \cdot r^{2}\cdot \left(1-\cos\theta \right)$$

(2) $$P_{Melt\;blending}=\left(S_{Melt\;blending}/S_{Sphere}\right)\times 100\%=\left(1-\cos\theta \right)\times 100\%$$

In contrast, fibers are pressed in an xy-plane for the composite produced through the VARI process, as shown in Figure 4 and Figure 9d. The probability of fiber occurrence within an angle θ in this space can be expressed as Equation (3):

(3) $$P_{VAMI}=\left(2\theta /180{}^{\circ}\right)\times 100\%$$

The relationship between $P_{Melt\;blending}$, $P_{VAMI}$, and θ is shown in Figure 9e. It is evident that $P_{VAMI}$ exceeds $P_{Melt\;blending}$ when θ is close to 0°. Therefore, more fibers are aligned closer to the printing direction when adopting the VARI process.

**Figure 10:**
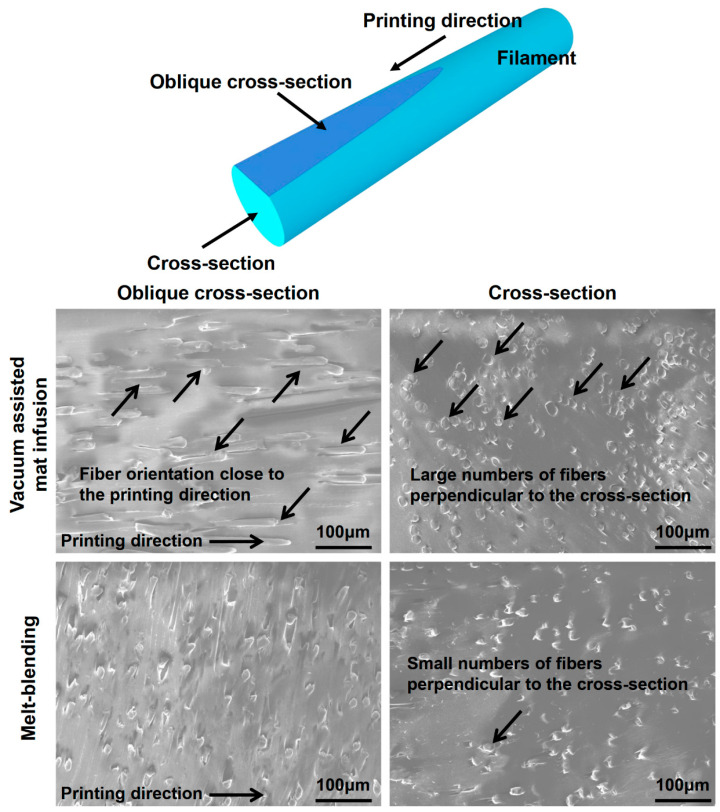
SEM morphology of filament produced using a vacuum-assisted resin infusion process and melt blending process before being printed.

**Figure 11:**
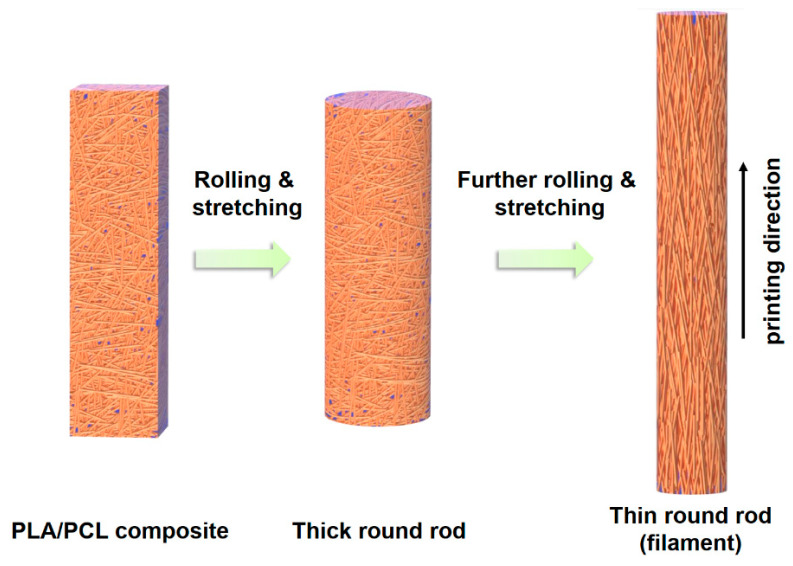
Schematic illustration of fiber orientation when the composite was rolled into filament during the VARI process.

As a result, compared to the composite prepared by melt blending, filaments produced from the VARI process yield a higher concentration of fibers oriented closer to the printing direction, thus further increasing the yield strength and Young’s modulus of the 3D printed strands. This is also supported by the experimental observations shown in Figure 10, where a large number of fibers were oriented close to the printing direction in the filament produced by the VARI process compared to the filament produced by the melt blending process. The rolling of PLA/PCL composites when they were made into filament may also promote more fibers to align toward the printing direction, as shown in Figure 11. However, rolling may be more effective for aligning the fibers in the PLA/PCL composites produced by the VARI process than the melt blending process (Figure 10).

### 3.4. Morphology and Mechanical Performance of 3D Printed Tissue Engineering Scaffolds

**Figure 12:**
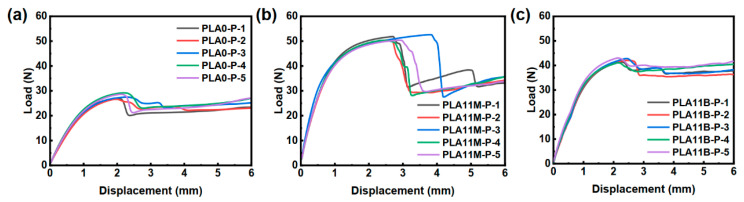
Typical force-displacement curves of 3D printed parts (**a**) PLA0-P, (**b**) PLA11M-P, and (**c**) PLA11B-P.

PLA/PCL composite with 11 wt% PLA fiber content was 3D printed into tissue engineering scaffolds [45]. Their load–displacement curves and mechanical performances are shown in Figure 12 and Table 5. As expected, compared to neat PCL (PLA0-P), the peak load and stiffness of the PLA11M-P were significantly increased by 84.22% and 143.28%, respectively. Due to the higher concentration of fibers aligned closer to the printing direction as described in Section 3.3, the percentage increase in stiffness and peak load of the 3D printed part produced through the VARI process (PLA11M-P) is higher than the melt blending (PLA11B-P) counterpart by 62.2% and 80.6%, respectively. As shown in Figure S5, compared with pure PCL (PLA0-P), both PLA11M-P and PLA11B-P show significant differences in mechanical properties (*p* < 0.001), and there is also a significant difference between PLA11M-P and PLA11B-P (*p* < 0.001). This significant improvement in the mechanical performance of the 3D printed parts shows the potential of adopting the highly fiber-oriented PLA/PCL composites produced by the VARI process for 3D printed tissue engineering technology.

## 4. Conclusions

In this work, vacuum vacuum-assisted resin infusion (VARI) process was developed to produce the PLA/PCL composite filament for 3D printed tissue engineering scaffolds with enhanced mechanical performance. The prepared PLA fiber mats exhibit good uniformity and interlocking characteristics, and the PLA/PCL composite can be produced with fewer defects through the VARI process as compared to the conventional hot-pressing and melt blending processes. The tensile strength and modulus of the PLA/PCL composite produced through the VARI process are significantly higher than the neat PCL.

The fibers are predominantly oriented on the same plane in the composite produced by the VARI process, which enables more fibers to align closer to the printing direction during 3D printing. This has further increased the mechanical performance of 3D printed strands and parts. At the same fiber content, the percentage of fibers with orientation angles within 30° of the 3D printed strands using the filaments produced using the VARI process is 41% higher than the printed strands using the filaments produced by the conventional melt blending process. And the percentage increase in yield strength and Young’s modulus of the 3D printed strands using the filaments by rolling and stretching of the PLA/PCL composite produced by the VARI process is 97.3% and 127.6% higher than the printed strands using the filaments produced by the conventional melt blending process. Porous tissue scaffold parts were printed using PLA/PCL composite with 11 wt% PLA fiber content and achieved an exceptional 84.2% and 143.3% increase in tensile peak load and stiffness compared to the 3D printed neat PCL counterpart. This composite material may be utilized to produce a new generation of tissue engineering scaffolds with higher mechanical performance and porosity while maintaining excellent biocompatibility and biodegradability.

## Figures and Tables

**Table 1:** Summary of main findings regarding various solvent systems employed in the preparation of PLA/PCL composites.

Materials	Preparation Method	Solvent System	References
PLLA fiber reinforced PCL composite scaffold	Fiber winding	N,N-dimethylacetamide	[25]
PLA fiber reinforced PCL composites	Electrospinning with hot-pressing	Chloroform and dimethylformamide (4:1 *w*/*w*)	[26]
PLA fiber reinforced PCL composites	Electrospinning with hot-pressing	Chloroform and N,N-dimethylacetamide (4:1 *v*/*v*)	[27]
PLA fiber reinforced PCL composite membrane	Electrospinning with annealing	Methylene chloride and N,N-dimethylformamide (7:3 *w*/*w*)	[29]
PLLA/PCL fibrous membrane	Electrospinning	Dichloromethane and dimethyl formamide (19:1 *w*/*w*)	[37]
PLA/PCL fibrous membrane	Electrospinning	Chloroform and acetone (3:1 *v*/*v*)	[38]
PLA/PCL fibrous membrane	Electrospinning	Chloroform and methanol (3:1 *v*/*v*)	[39]
PLA/PCL fibrous membrane	Electrospinning	Chloroform and N,N-dimethylacetamide (90~70:10~30 *v*/*v*)	[40]

**Table 2:** Material ratios of raw PLA/PCL composites.

Designations	Composition by Weight (%)	
	PCL	PLA
PCL	100	0
PLA/PCL-6	94	6
PLA/PCL-11	89	11
PLA/PCL-16	84	16

**Table 3:** Yield strength and Young′s modulus of PCL and PLA/PCL composites via VARI process.

Designations	Yield Strength (MPa)	Percentage Increase (%)	Young’s Modulus (MPa)	Percentage Increase (%)	Elongation at Break (%)
PCL	13.00 ± 0.30	-	281.25 ± 5.55	-	705.12 ± 12.67
PLA/PCL-6	14.24 ± 0.84	9.54	390.24 ± 20.14	38.75	910.34 ± 16.87
PLA/PCL-11	16.10 ± 0.32	23.85	553.22 ± 21.19	96.70	50.54 ± 2.65
PLA/PCL-16	19.30 ± 1.17	48.46	676.76 ± 27.63	140.63	54.01 ± 3.98

**Table 4:** Yield strength, Young’s modulus and coefficient of variation (C.V.) of 3D printed neat PCL (PLA0-S), 3D printed PLA/PCL strands with 11 wt% PLA fiber content produced by the vacuum assisted resin infusion process (PLA11M-S), and 3D printed PLA/PCL strands with 11 wt% PLA fiber content produced by the melt blending process (PLA11B-S).

Designation	Yield Strength (MPa)	C.V.	Percentage Increase (%)	Young’s Modulus (MPa)	C.V.	Percentage Increase (%)
PLA0-S	13.48 ± 0.10	0.74	-	279.15 ± 10.81	3.87	-
PLA11M-S	19.24 ± 0.92	4.78	42.73	693.56 ± 48.35	6.97	148.45
PLA11B-S	16.40 ± 0.53	3.23	21.66	461.18 ± 15.83	3.43	65.21

**Table 5:** Peak load, stiffness, coefficient of variation (C.V.) of 3D printed parts with neat PLA (PLA0-P), 3D printed PLA/PCL parts with 11 wt% PLA fiber content produced by the vacuum assisted resin infusion process (PLA11M-P) and 3D printed PLA/PCL strands with 11 wt% PLA fiber content produced by the melt blending process (PLA11B-P).

Designation	Peak Load (N)	C.V. (%)	Percentage Increase (%)	Stiffness (N/m)	C.V. (%)	Percentage Increase (%)
PLA0-P	27.75 ± 1.08	3.89	-	38.52 ± 1.04	2.70	-
PLA11M-P	51.12 ± 1.03	2.01	84.22	93.71 ± 2.05	2.19	143.28
PLA11B-P	42.16 ± 0.87	2.06	51.93	69.08 ± 3.93	5.69	79.33

## Data Availability

The original contributions presented in this study are included in the article and Supplementary Materials. Further inquiries can be directed to the corresponding authors.

## Supplementary Materials

The following supporting information can be downloaded at: https://www.mdpi.com/article/10.3390/polym17152138/s1, Figure S1. DSC curves of the second heating run for PCL, PLA fibers, PCL exposed to 120 °C (PCL-120), and PLA fibers exposed to 120 °C (PLA-120). Figure S2. Optical images of PLA fibers. (a,b) Raw fibers. (c,d) Fibers separated from the composite prepared by melt blending. Figure S3. Yield strength and Young′s modulus of PLA0-S, PLA11M-S, and PLA11B-S. * *p* < 0.05, ** *p* < 0.01, and *** *p* < 0.001. Figure S4: Schematic diagram for calculating fiber orientation angle. Figure S5. Peak load and stiffness of PLA0-S, PLA11M-S, and PLA11B-S. * *p* < 0.05, ** *p* < 0.01, and *** *p* < 0.001.
