# Supplementary material for: Highly-Oriented Polylactic Acid Fiber Reinforced Polycaprolactone Composite Produced by Infused Fiber Mat Process for 3D Printed Tissue Engineering Technology
Source: Polymers (Basel). 2025 Aug 5;17(15):2138. doi: 10.3390/polym17152138 (PMC12349036; doi:10.3390/polym17152138)
Supplement: Supplementary file 1 [file polymers-17-02138-s001.zip › polymers-3713867-supplementary.pdf]

Supplementary Materials

For DSC measurements, a PerkinElmer DSC6000 instrument was used. Thermal cycles were composed of three cycles: a first heating scan, from 25°C to 200°C, to erase the thermal history of the sample; a cooling scan from 200°C to 25°C and finally, another heating scan from 25°C to 200°C. The three scans were performed with a heating rate of 10 °C/min under nitrogen purge (50 mL/min). The degree of crystallinity of the PLA fiber was calculated by the following equations:

$$\chi_c(\%) = \frac{\Delta H_m - \Delta H_{cc}}{\Delta H_m^{\circ}} \times 100\% \tag{1}$$

where ΔH_m° is the enthalpy value of 100% crystalline PLA, 93 J/g [1]. Heat of fusion (ΔH_m) and enthalpy of cold crystallization (ΔH_{cc}) is the enthalpy corresponding to the second heating scan.

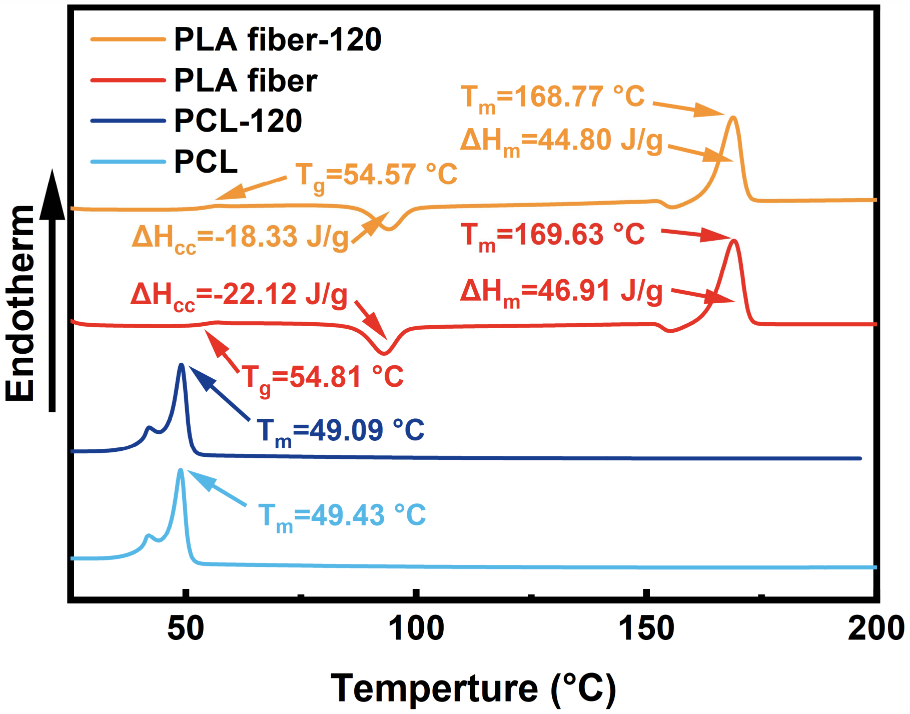

Figure S1. DSC curves of the second heating run for PCL, PLA fibers, PCL exposed to 120°C (PCL-120), and PLA fibers exposed to 120°C (PLA-120).

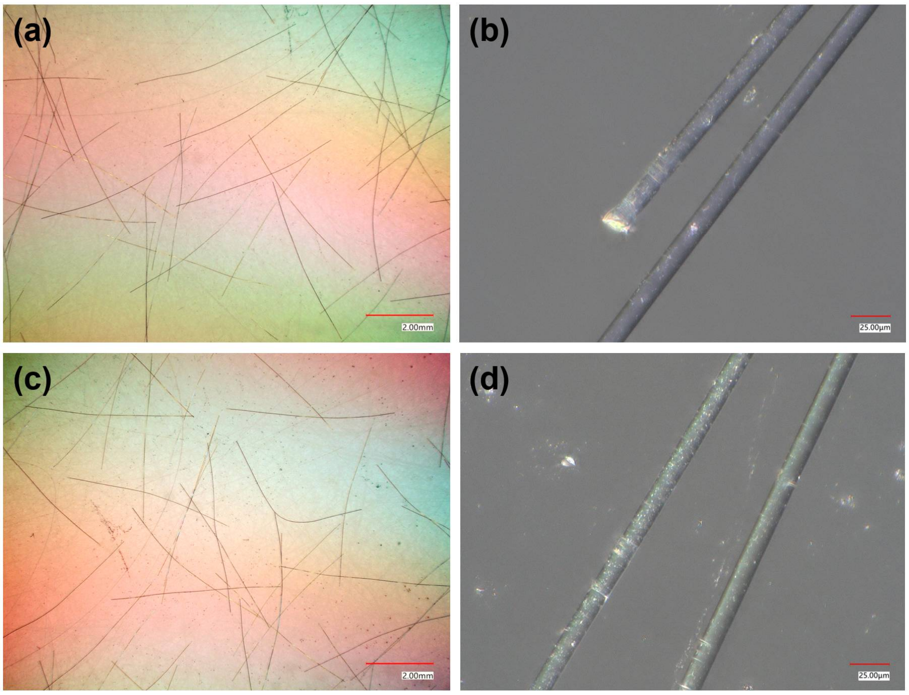

Figure S2. Optical images of PLA fibers. (a-b) Raw fibers, (c-d) Fibers separated from the composite prepared through melt blending.

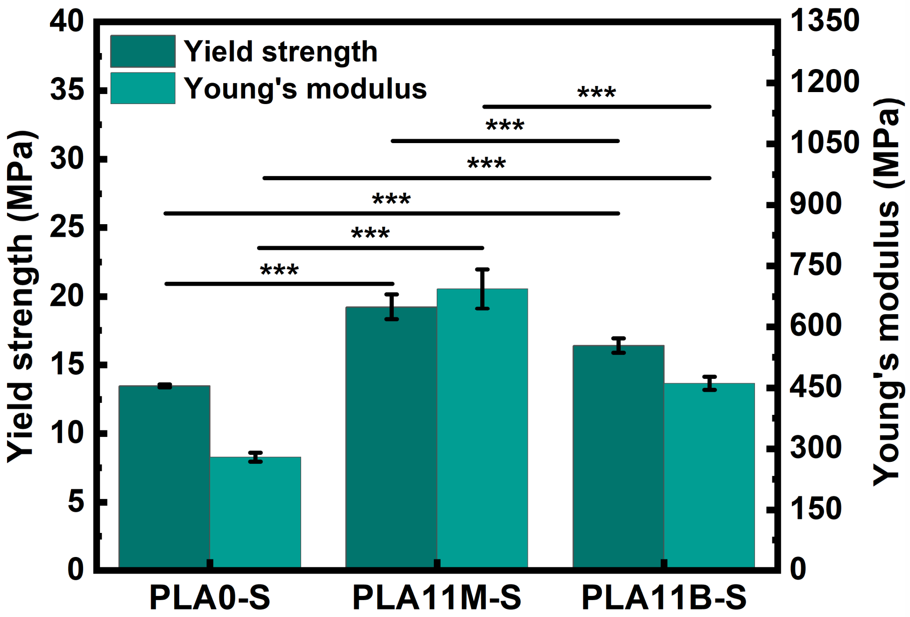

Figure S3. Yield strength and Young's modulus of PLA0-S, PLA11M-S, and PLA11B-S. *** $p < 0.001$.

As shown in Fig. S4, the ABCD plane is the cross-section of the sample (i.e., the observation plane of the SEM), and the straight-line EF is a normal to this cross-section

and also the axial direction of the sample. A fiber passes through this observation plane, with GH the axis of the fiber. The red area enclosed by the red dashed line is the cross-sectional area of the fiber observed on the observation plane, which is an ellipse. O' is the center of this ellipse, IN is the major axis *a* of the ellipse, and PQ is the minor axis *b* of the ellipse. By extending the edges of the ellipse, the circular cross-section of the fiber (i.e., the blue area) is obtained, where O is the center of the circle and MN is the diameter of the circle. Assuming that the angle between the fiber and the axial direction of the sample is α, that is, the angle between the straight-line EF and GH, and that the angle between the fiber and the ABCD plane is β, then the following relationship holds:

$$\alpha + \beta = 90° \tag{1}$$

$$\beta = \arcsin\left(\frac{MN}{IN}\right) = \arcsin\left(\frac{b}{a}\right) \tag{2}$$

$$\alpha = 90° - \arcsin\left(\frac{b}{a}\right) \tag{3}$$

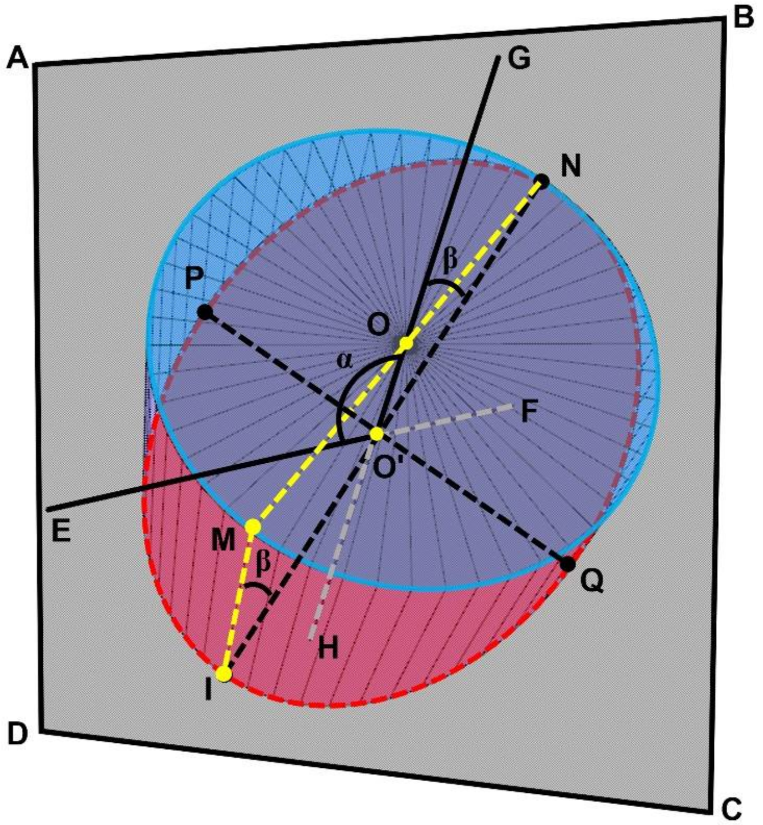

Figure S4. Schematic diagram for calculating fiber orientation angle.

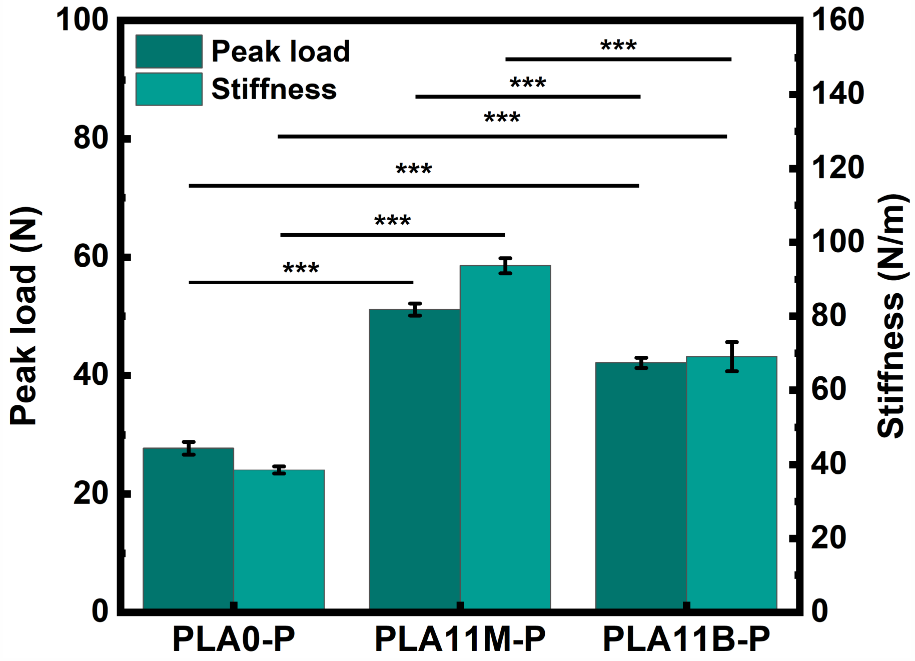

Figure S5. Peak load and stiffness of PLA0-S, PLA11M-S, and PLA11B-S. *** $p < 0.001$.

Reference

[1] Botlhoko, O.J.; Ramontja, J.; Ray, S.S. A new insight into morphological, thermal, and mechanical properties of melt-processed polylactide/poly(ε-caprolactone) blends. *Polym. Degrad. Stabil.* **2018**, 154, 84-95.

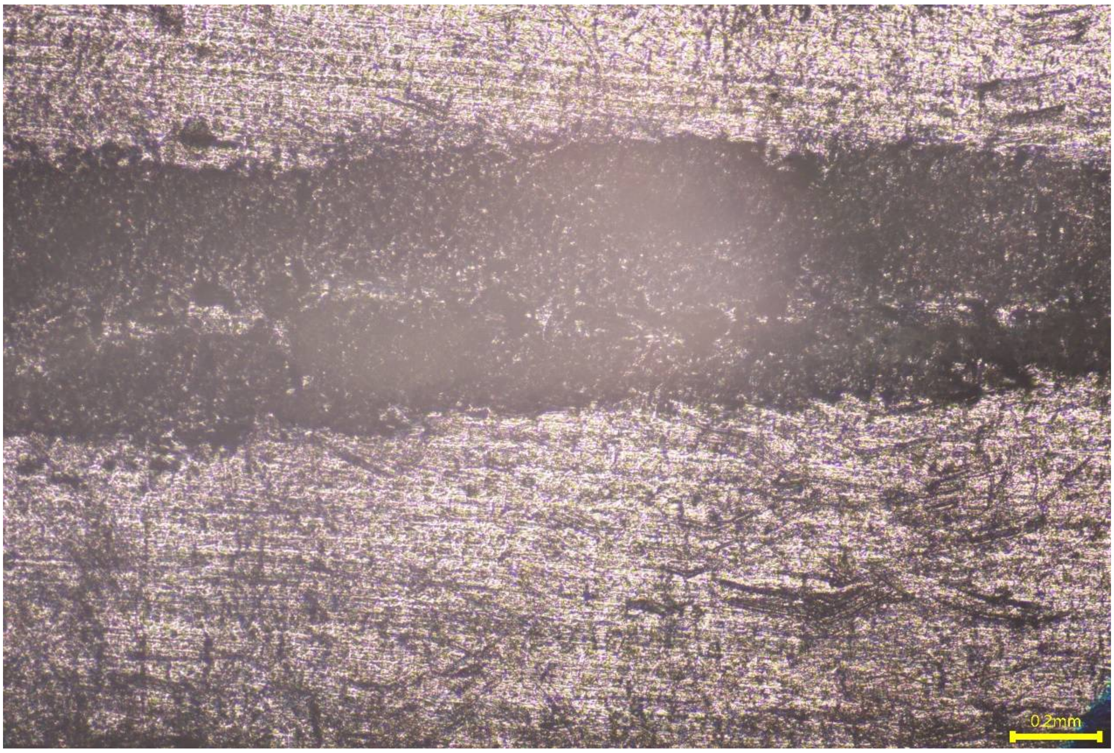

Corresponding to Figure 3c in the main article.

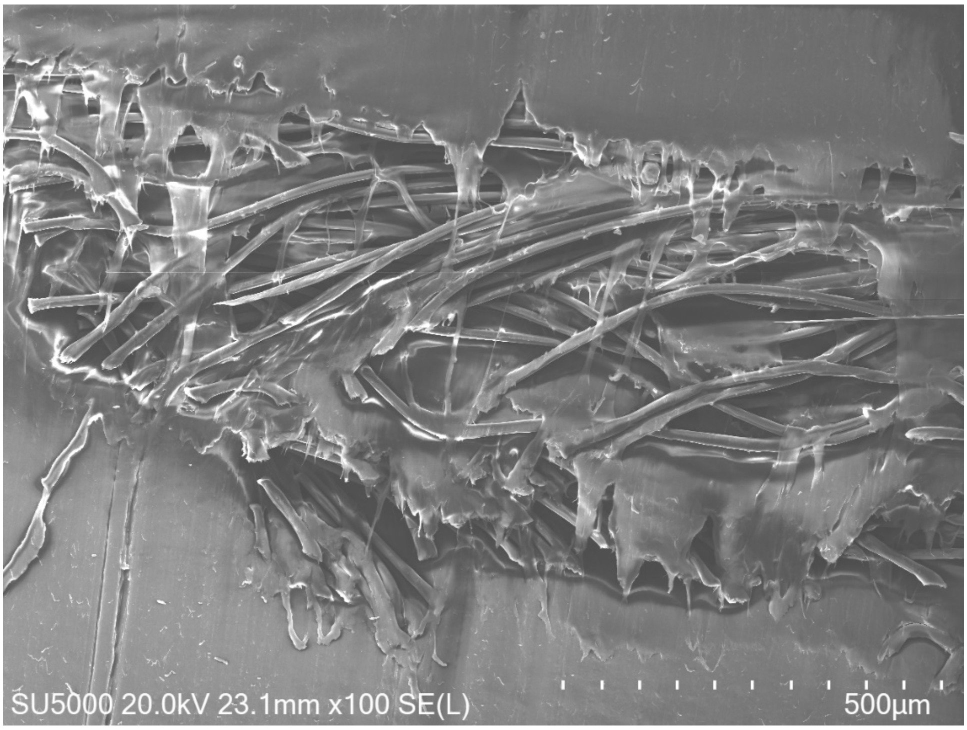

Corresponding to Figure 3d in the main article.

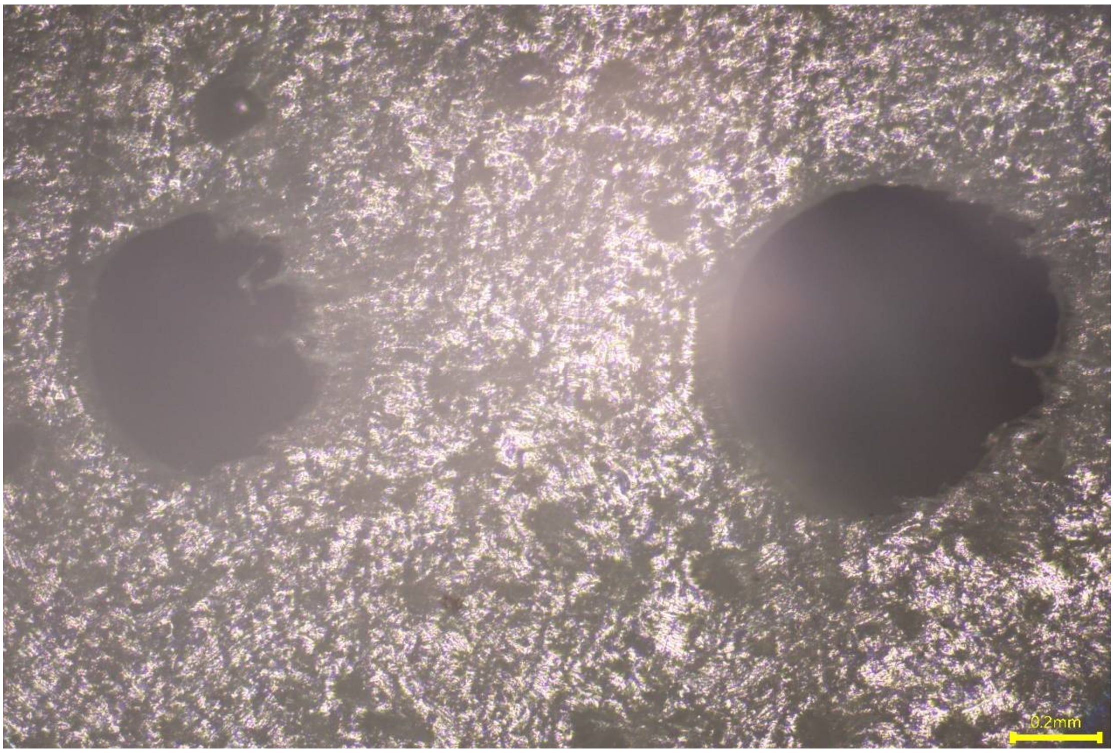

Corresponding to Figure 3g in the main article.

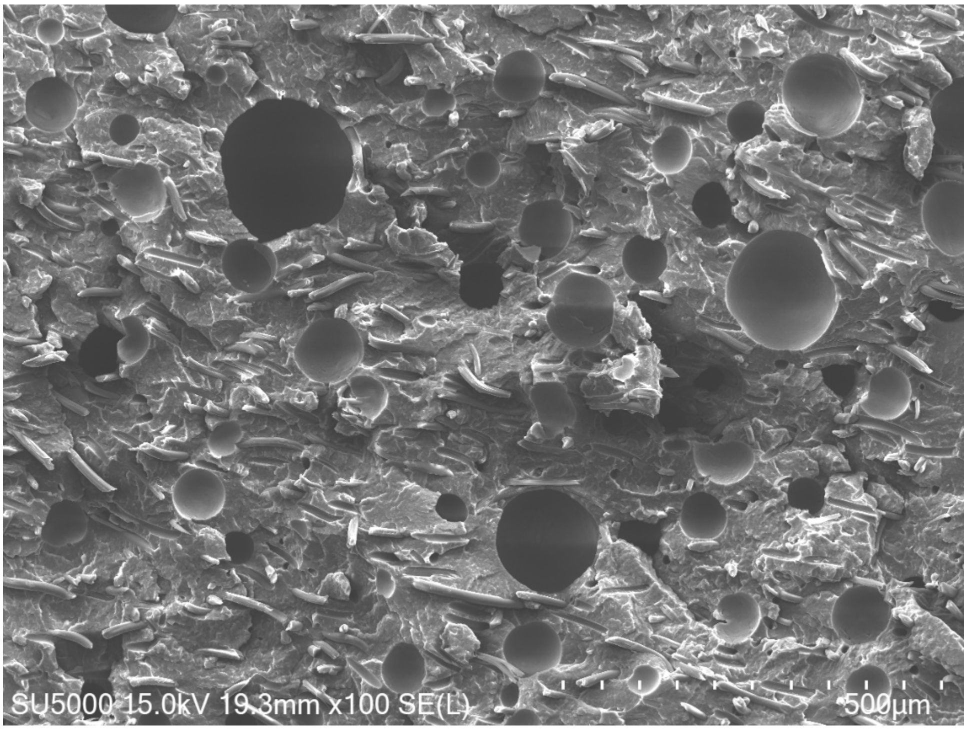

Corresponding to Figure 3h in the main article.

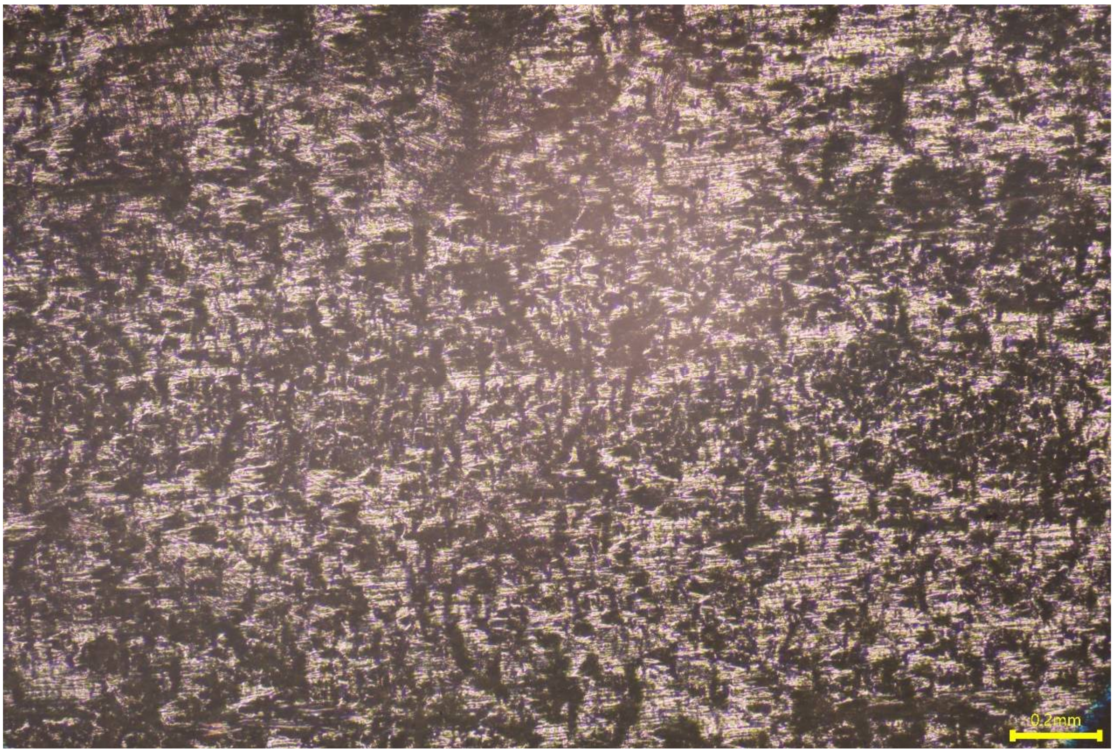

Corresponding to Figure 3k in the main article.

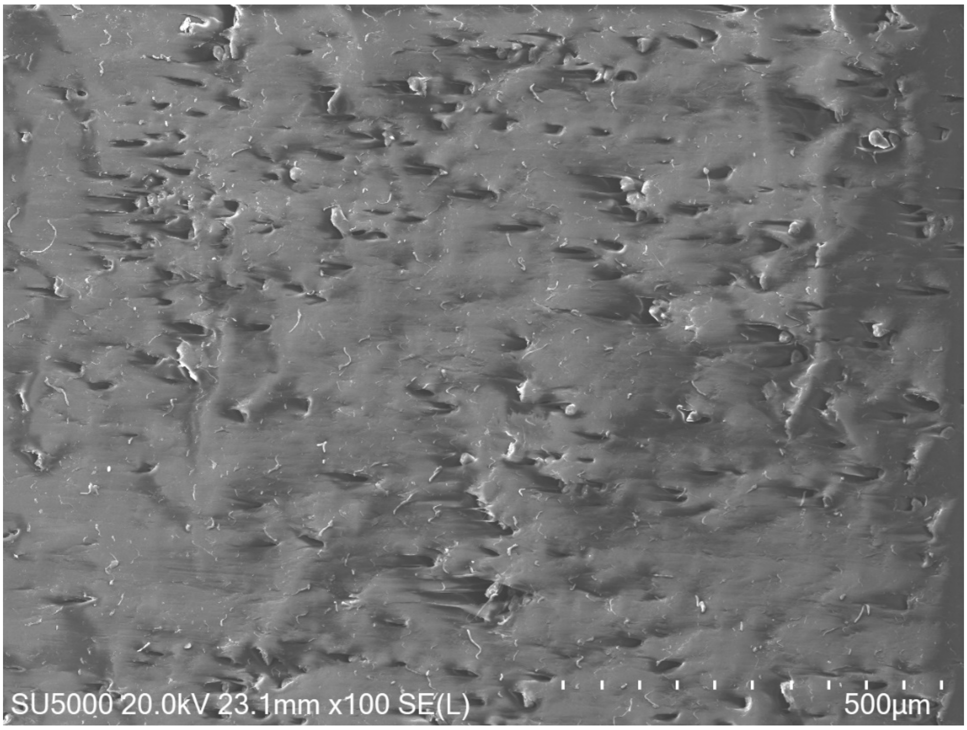

Corresponding to Figure 3l in the main article.

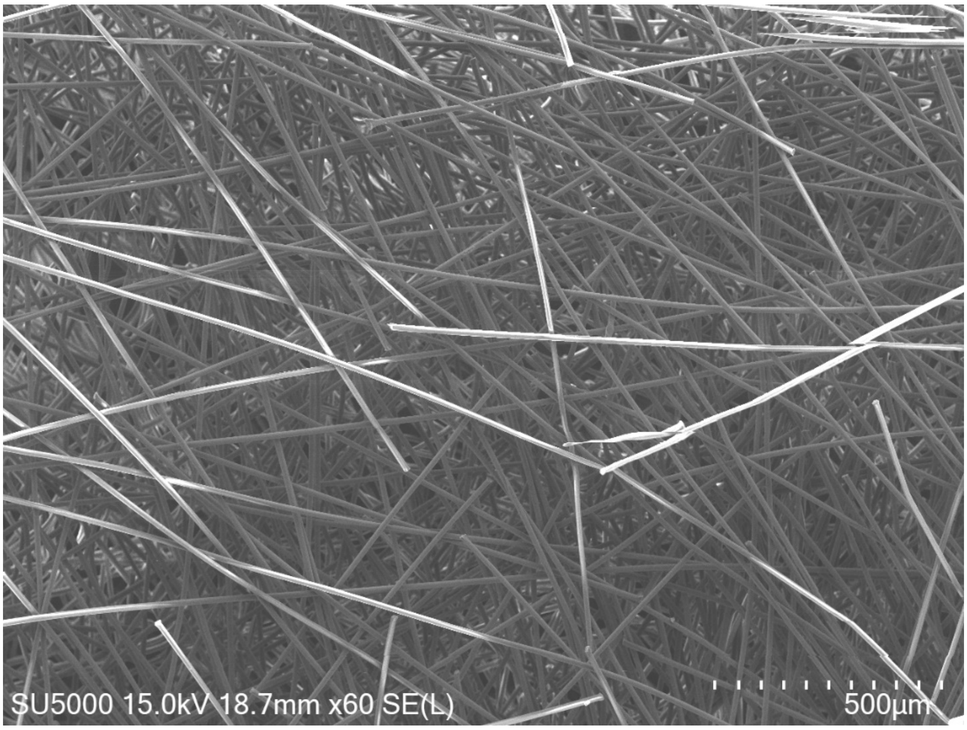

Corresponding to Figure 4b in the main article.

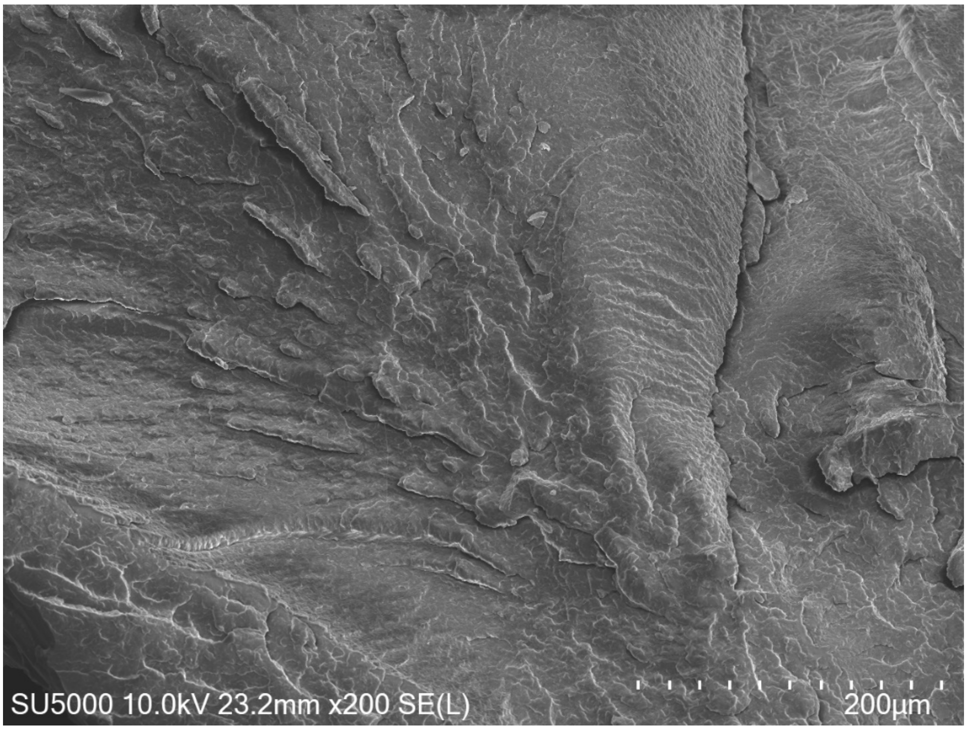

Corresponding to Figure 6a in the main article.

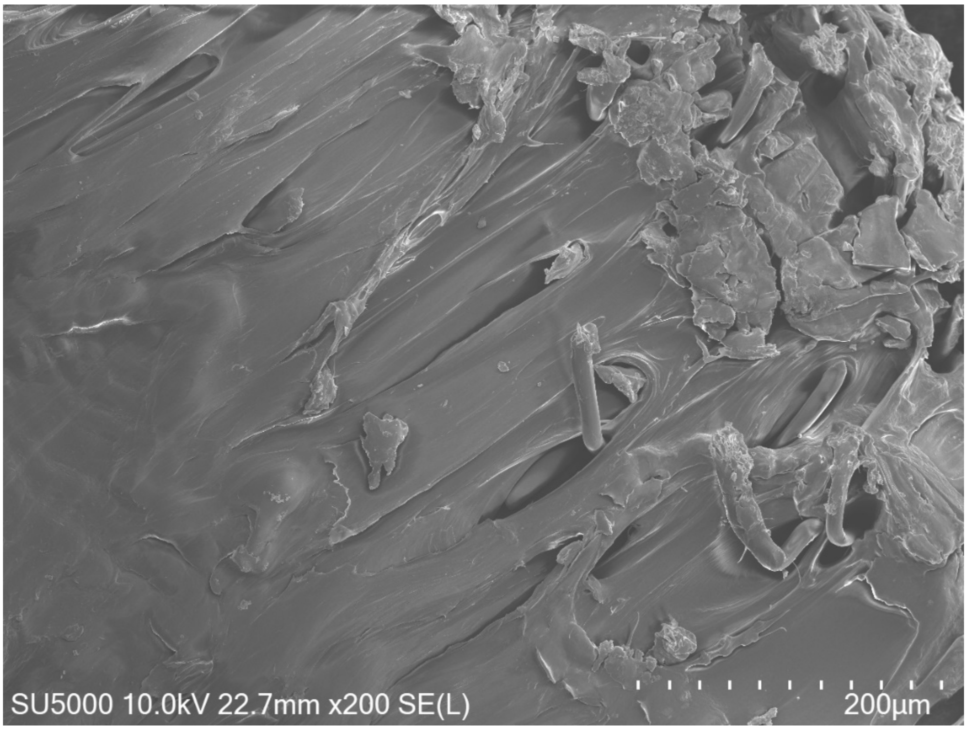

Corresponding to Figure 6b in the main article.

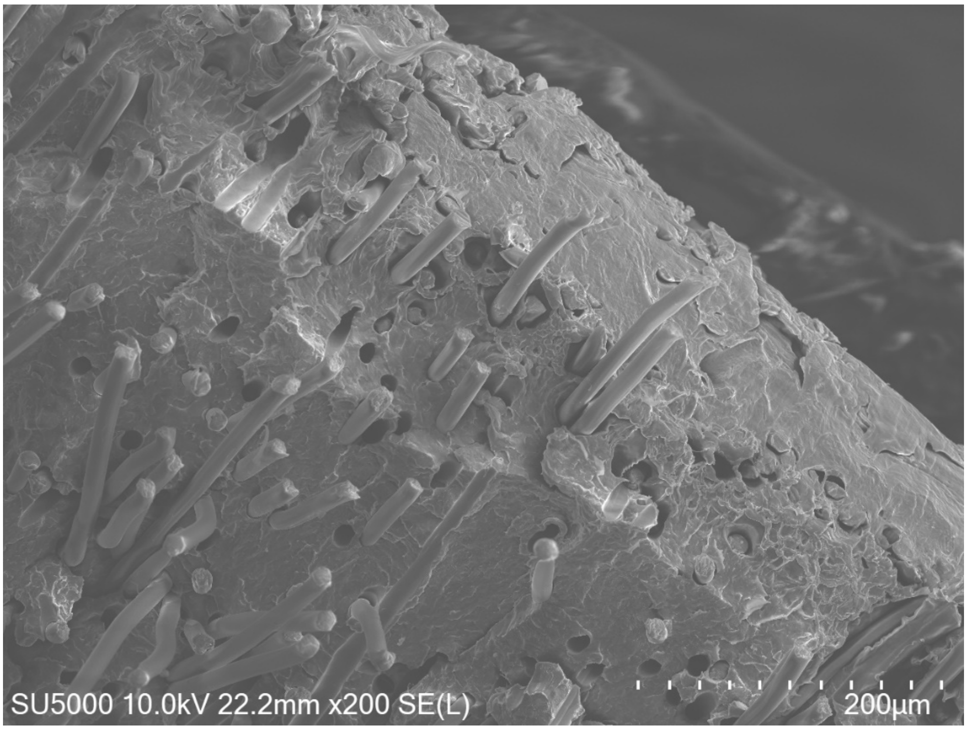

Corresponding to Figure 6c in the main article.

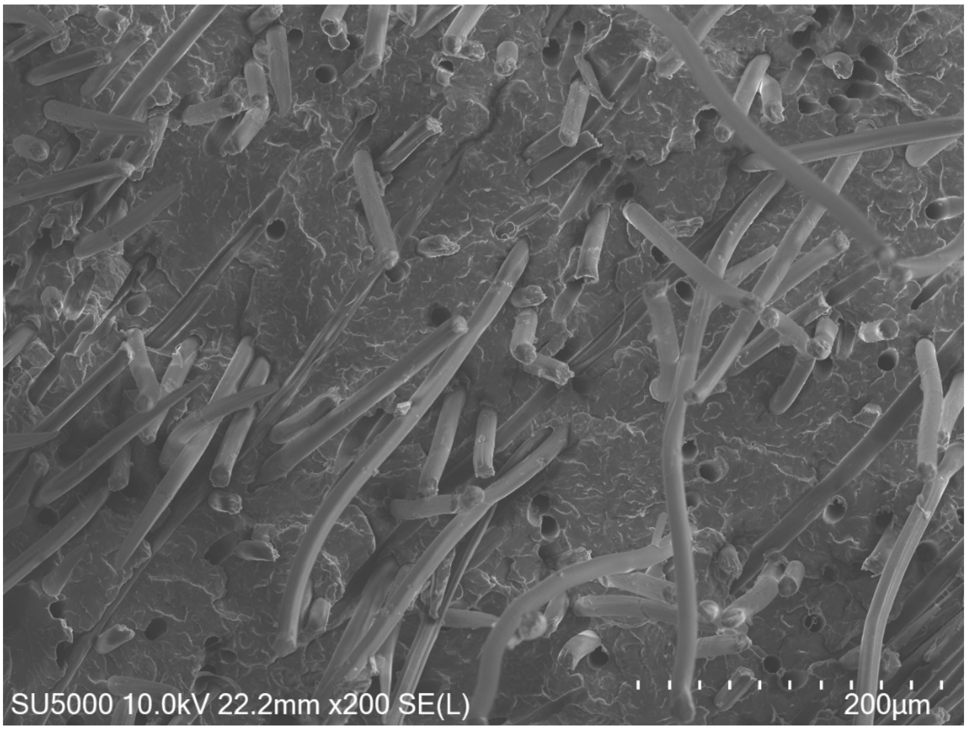

Corresponding to Figure 6d in the main article.

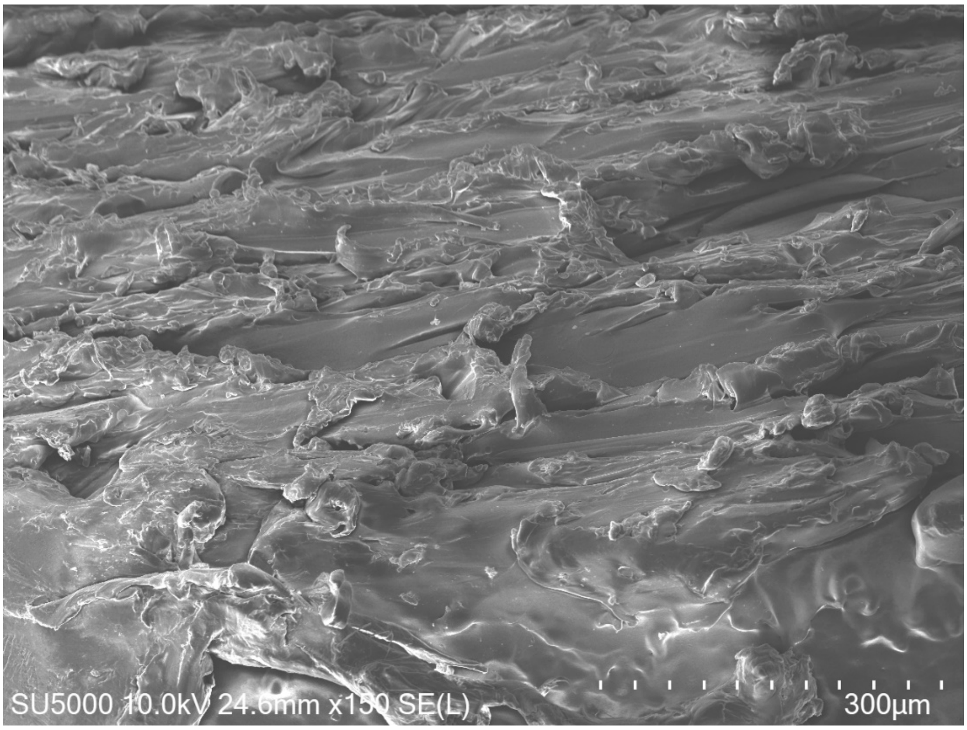

Corresponding to Figure 6e in the main article.

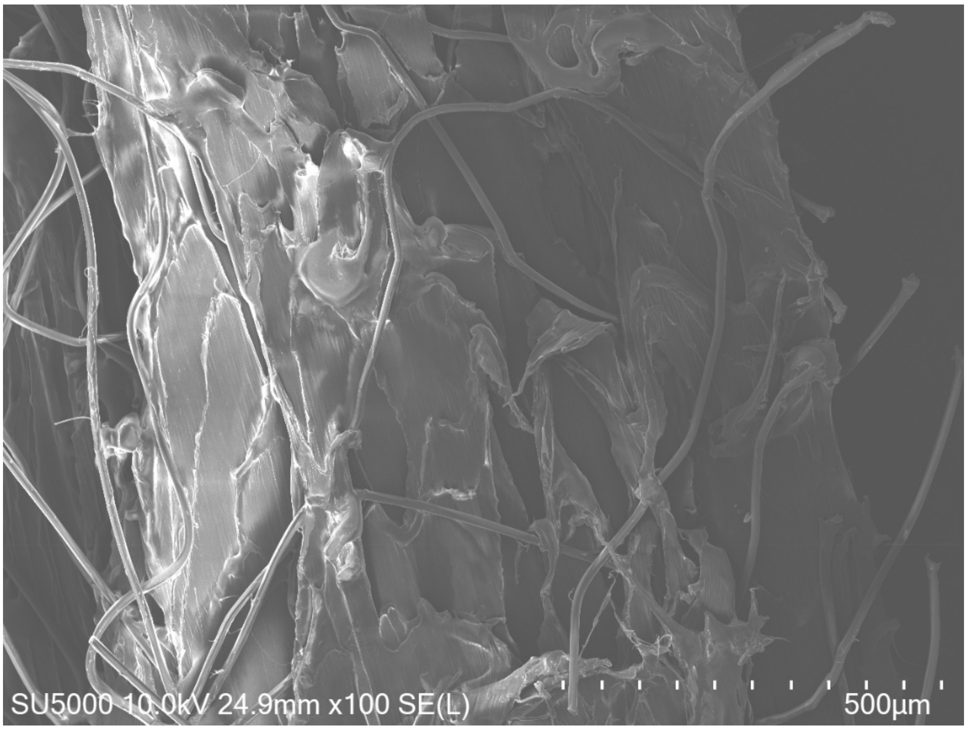

Corresponding to Figure 6f in the main article.

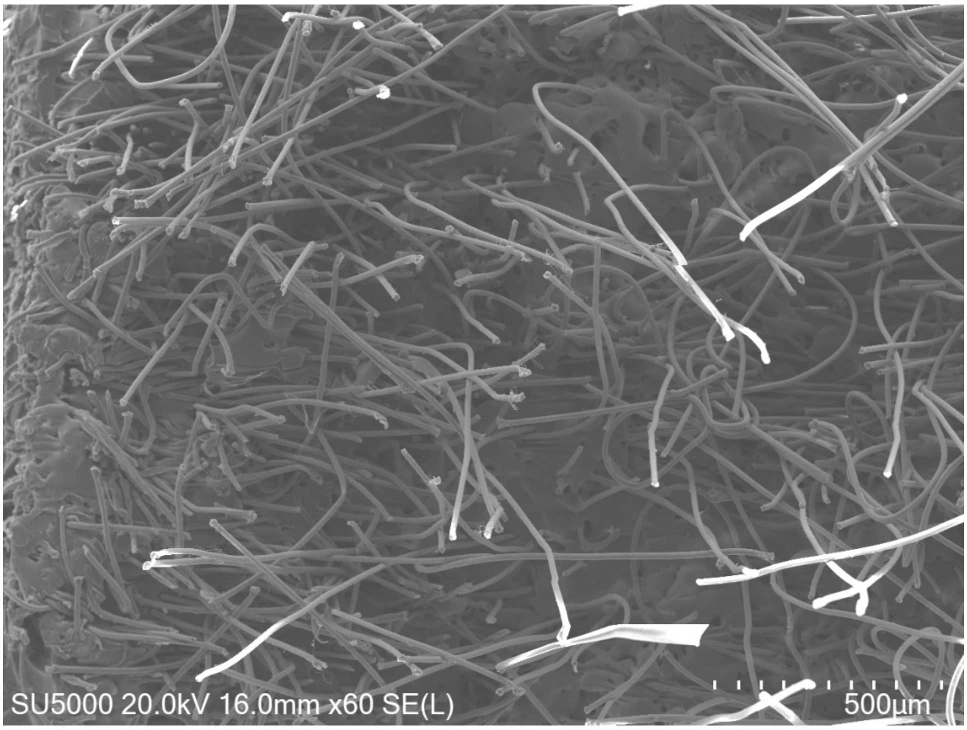

Corresponding to Figure 6g in the main article.

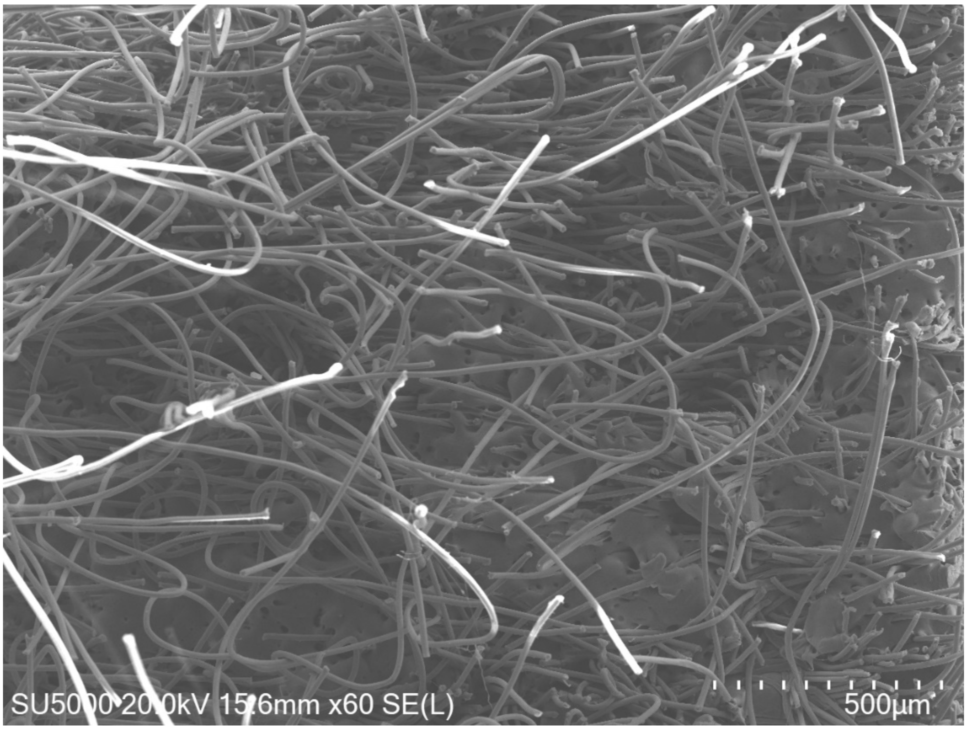

Corresponding to Figure 6h in the main article.

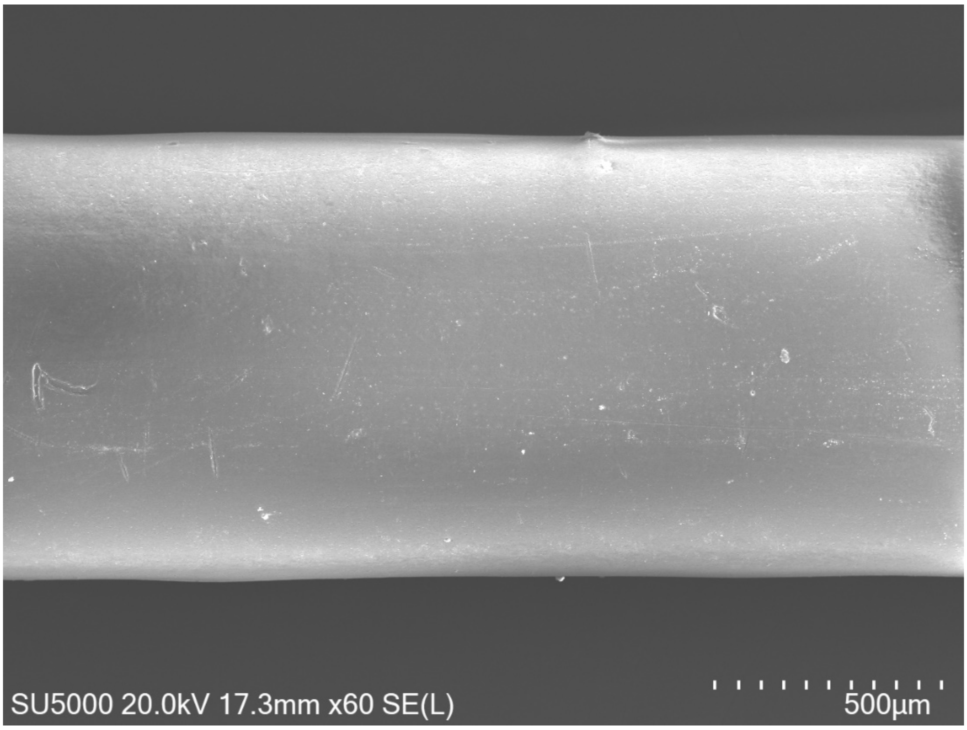

Corresponding to Figure 8a in the main article.

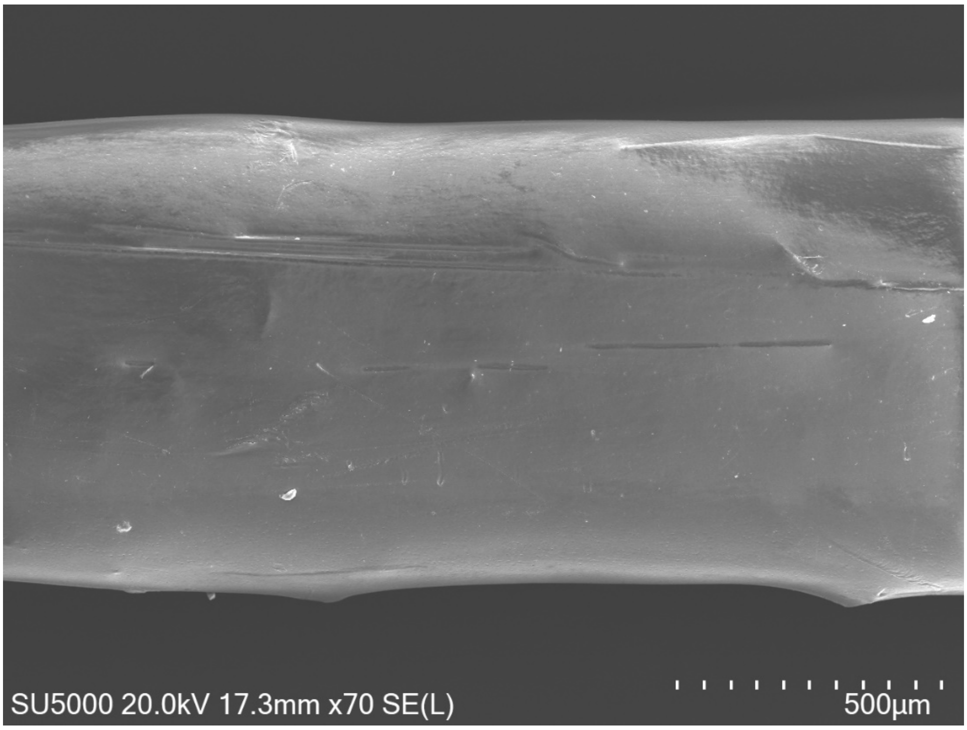

Corresponding to Figure 8b in the main article.

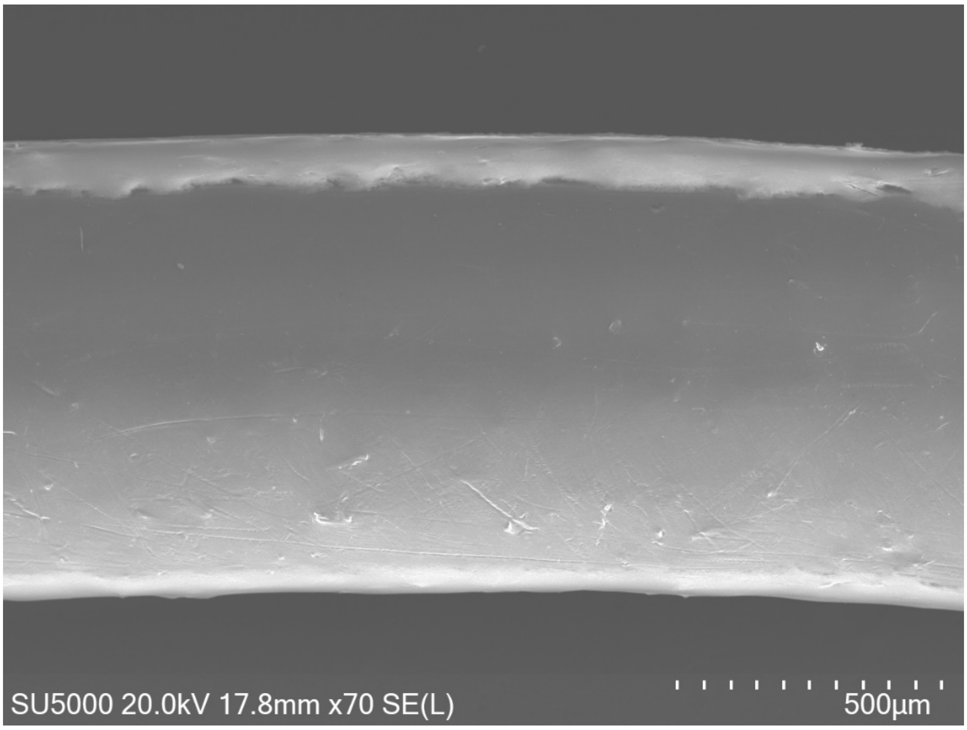

Corresponding to Figure 8c in the main article.

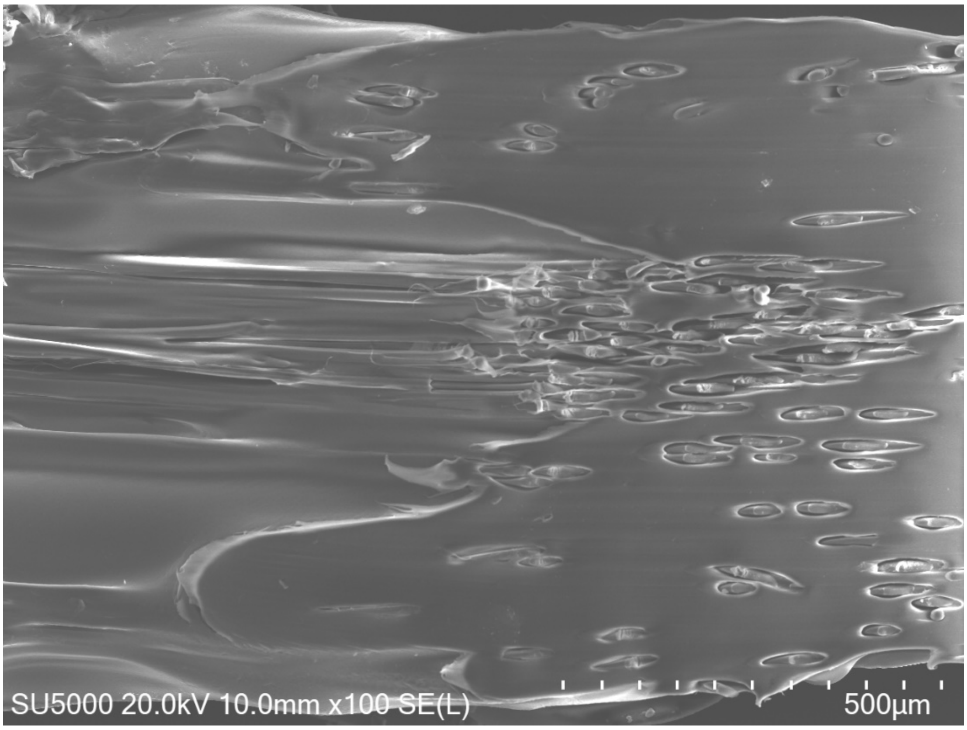

Corresponding to Figure 8d in the main article.

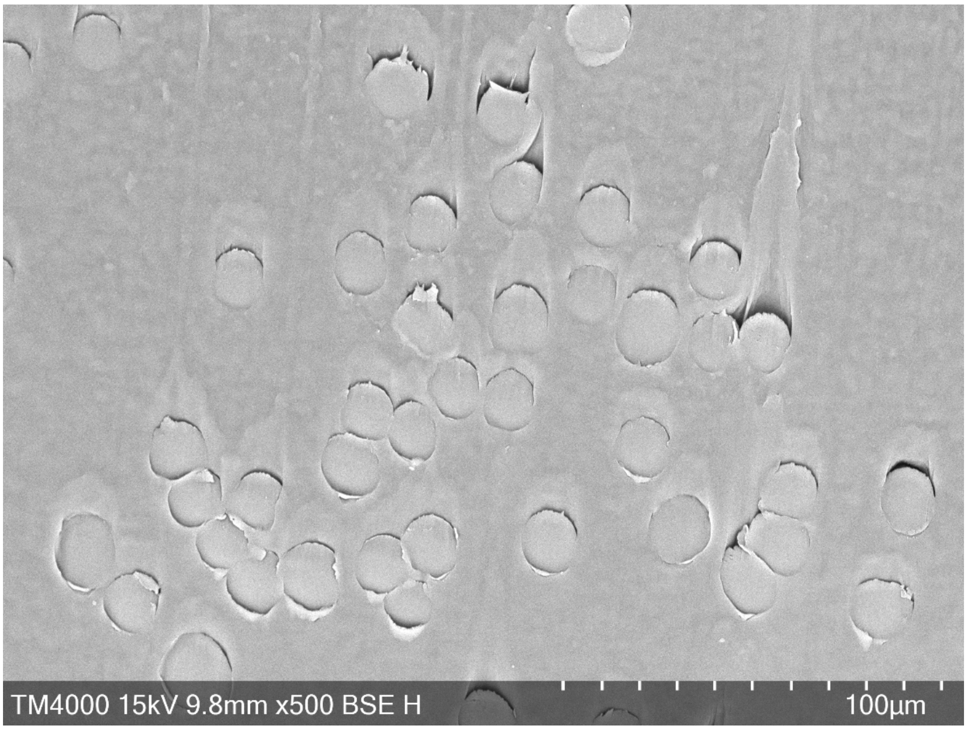

Corresponding to Figure 8e in the main article.

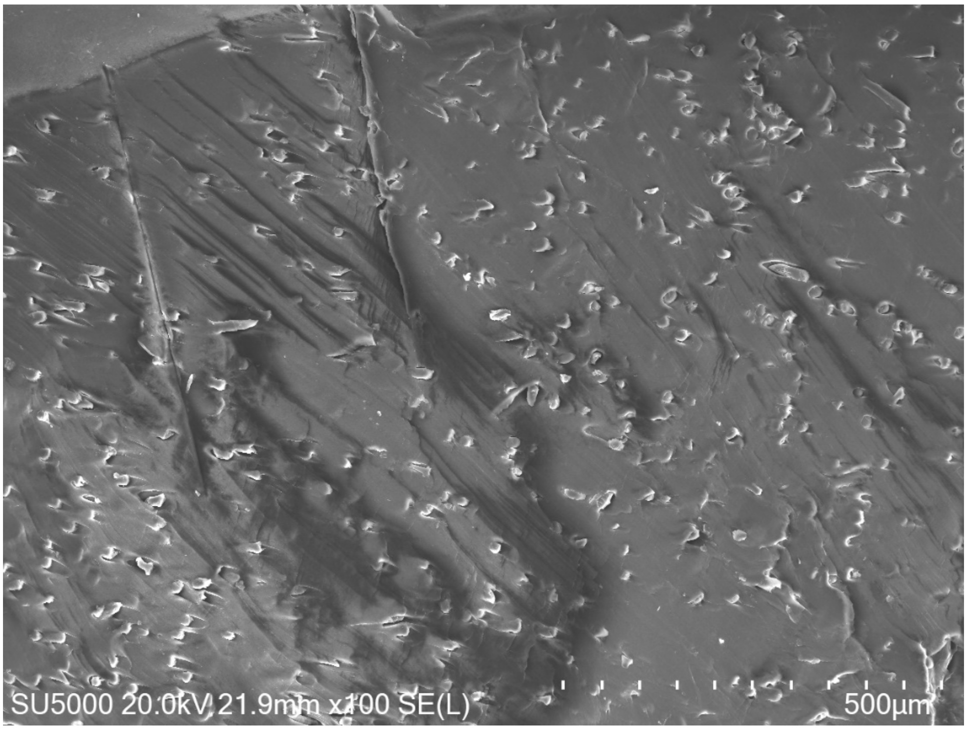

Corresponding to Figure 8g in the main article.

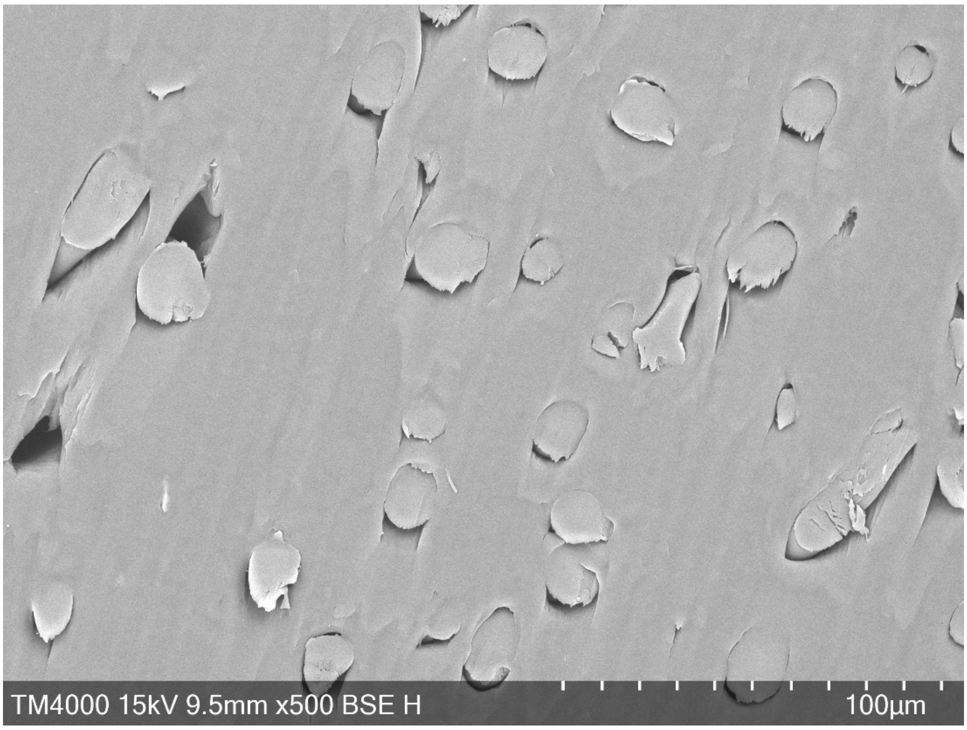

Corresponding to Figure 8h in the main article.

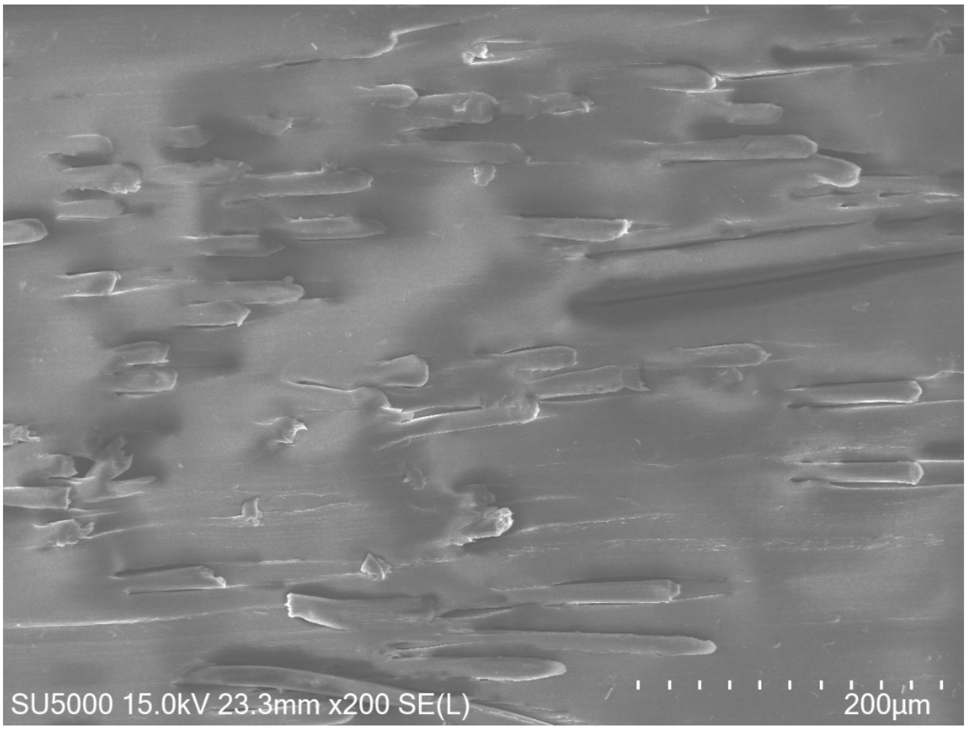

Corresponding to the SEM in the upper left corner of Figure 10 in the main article.

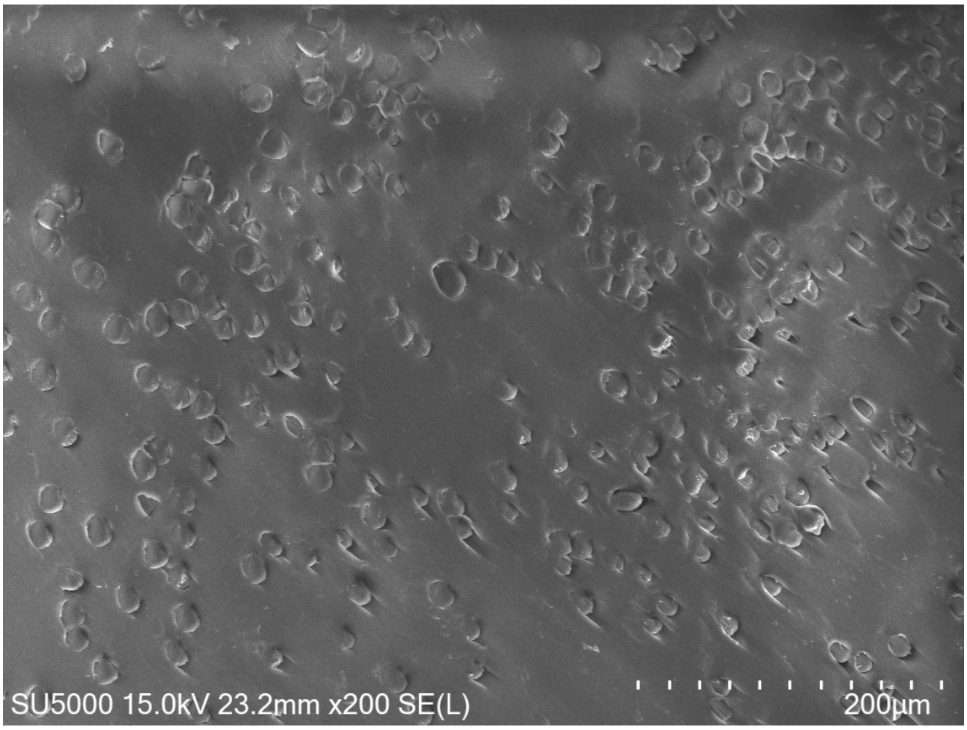

Corresponding to the SEM in the upper right corner of Figure 10 in the main article.

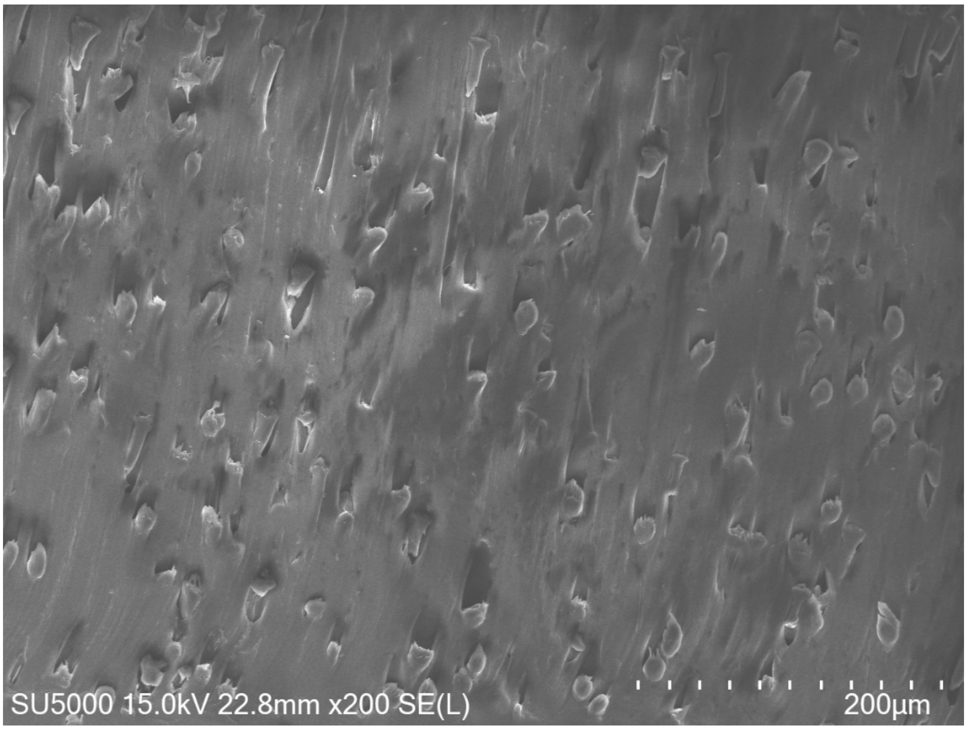

Corresponding to the SEM in the bottom left corner of Figure 10 in the main article.

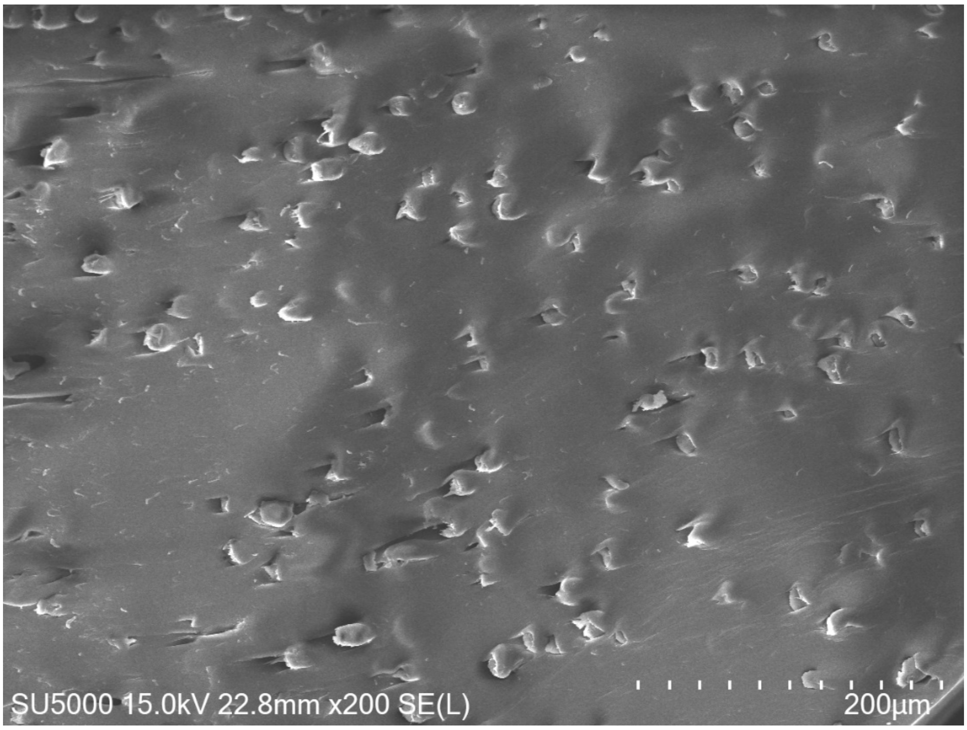

Corresponding to the SEM in the bottom right corner of Figure 10 in the main article.

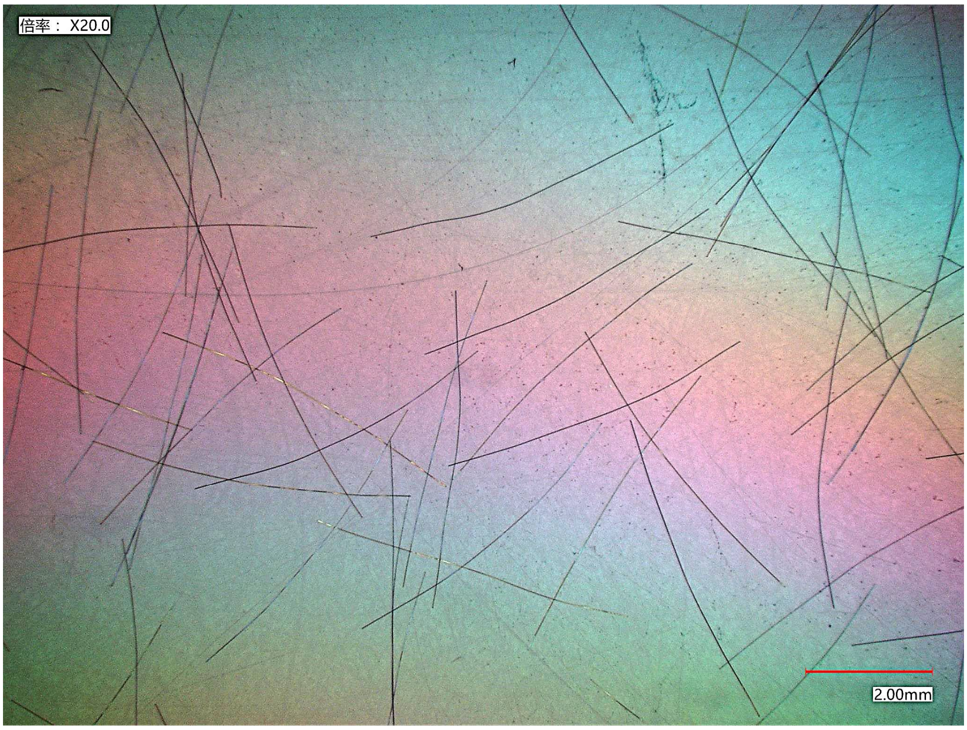

Corresponding to Figure S2(a) in the supplementary materials.

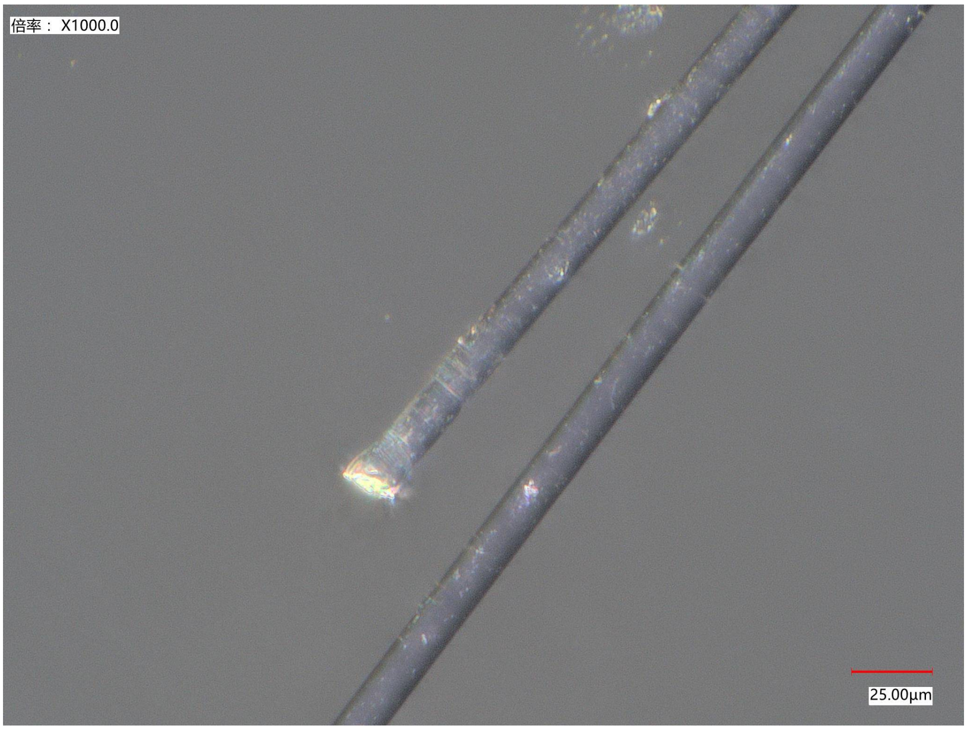

Corresponding to Figure S2(b) in the supplementary materials.

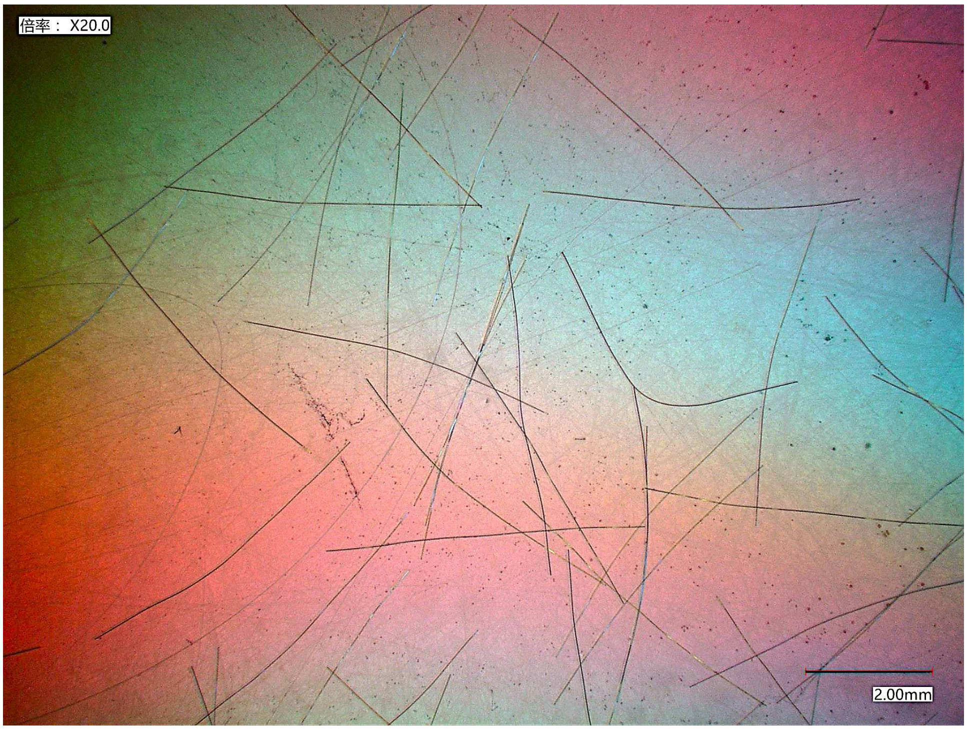

Corresponding to Figure S2(c) in the supplementary materials.

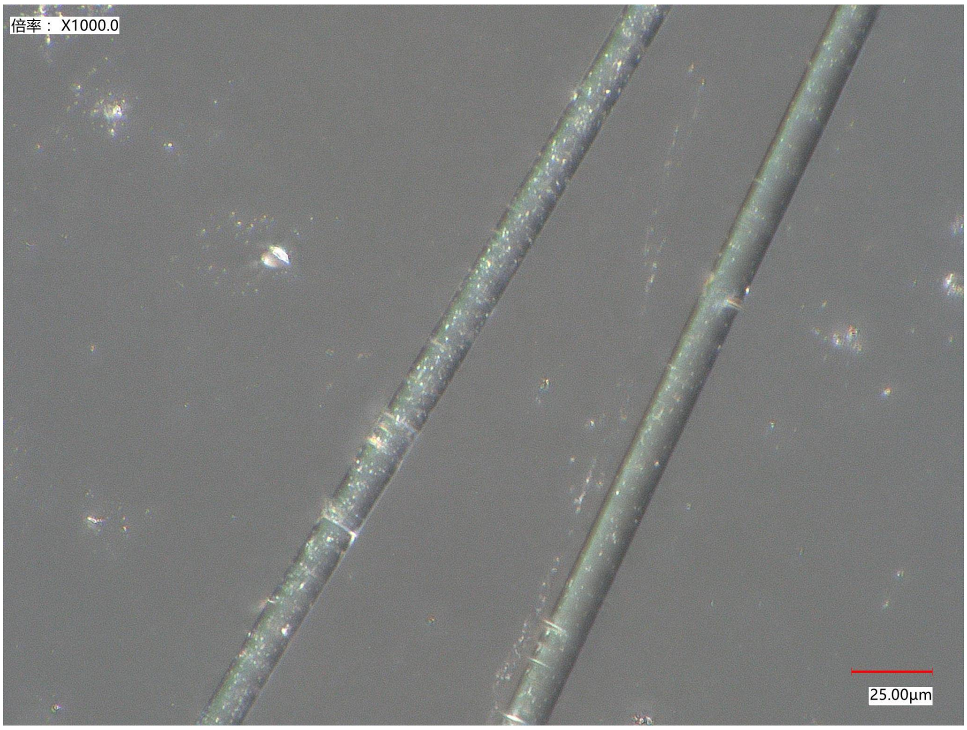

Corresponding to Figure S2(d) in the supplementary materials.
